# An Epigenetic Signature in Peripheral Blood Associated with the Haplotype on 17q21.31, a Risk Factor for Neurodegenerative Tauopathy

**DOI:** 10.1371/journal.pgen.1004211

**Published:** 2014-03-06

**Authors:** Yun Li, Jason A. Chen, Renee L. Sears, Fuying Gao, Eric D. Klein, Anna Karydas, Michael D. Geschwind, Howard J. Rosen, Adam L. Boxer, Weilong Guo, Matteo Pellegrini, Steve Horvath, Bruce L. Miller, Daniel H. Geschwind, Giovanni Coppola

**Affiliations:** 1 Department of Psychiatry and Semel Institute for Neuroscience and Human Behavior, David Geffen School of Medicine, University of California Los Angeles, Los Angeles, California, United States of America; 2 Interdepartmental Program in Bioinformatics, University of California Los Angeles, Los Angeles, California, United States of America; 3 Program in Neurogenetics, Department of Neurology, David Geffen School of Medicine, University of California Los Angeles, Los Angeles, California, United States of America; 4 Memory and Aging Center/Sandler Neurosciences Center, University of California San Francisco, San Francisco, California, United States of America; 5 Bioinformatics Division and Center for Synthetic & Systems Biology, TNLIST, Tsinghua University, Beijing, China; 6 Department of Molecular, Cell and Developmental Biology, David Geffen School of Medicine, University of California Los Angeles, Los Angeles, California, United States of America; 7 Departments of Biostatistics and Human Genetics, David Geffen School of Medicine, University of California Los Angeles, Los Angeles, California, United States of America; The University of North Carolina at Chapel Hill, United States of America

## Abstract

Little is known about how changes in DNA methylation mediate risk for human diseases including dementia. Analysis of genome-wide methylation patterns in patients with two forms of tau-related dementia – progressive supranuclear palsy (PSP) and frontotemporal dementia (FTD) – revealed significant differentially methylated probes (DMPs) in patients versus unaffected controls. Remarkably, DMPs in PSP were clustered within the 17q21.31 region, previously known to harbor the major genetic risk factor for PSP. We identified and replicated a dose-dependent effect of the risk-associated H1 haplotype on methylation levels within the region in blood and brain. These data reveal that the H1 haplotype increases risk for tauopathy via differential methylation at that locus, indicating a mediating role for methylation in dementia pathophysiology.

## Author Summary

Progressive supranuclear palsy (PSP) and frontotemporal dementia (FTD) are two neurodegenerative diseases linked, at the pathologic and genetic level, to the microtubule associated protein tau. We studied epigenetic changes (DNA methylation levels) in peripheral blood from patients with PSP, FTD, and unaffected controls. Analysis of genome-wide methylation patterns revealed significant differentially methylated probes in patients versus unaffected controls. Remarkably, differentially methylated probes in PSP vs. controls were preferentially clustered within the 17q21.31 region, previously known to harbor the major genetic risk factor for PSP. We identified and replicated a dose-dependent effect of the risk-associated H1 haplotype on methylation levels within the region in independent datasets in blood and brain. These data reveal that the H1 haplotype increases risk for tauopathy via differential methylation, indicating a mediating role for methylation in dementia pathophysiology.

## Introduction

Epigenetics is one of the most rapidly expanding fields in biology, and is uncovering additional levels of complexity in the human genome, including DNA methylation, histone modifications, and intra- and inter-chromosomal interactions mediated by chromatin proteins [1], [2]. Changes in methylation represent a key area where environmental factors can modify or interact with inherited genetic factors (DNA sequence) to alter the functional output of the genome. Disease-causing genes involved in epigenetic modifications have been identified, most notably for neurodevelopmental disorders such as Rett syndrome [3]. A very limited number of studies have addressed specific epigenetic modifications relevant to neurological diseases and dementia (reviewed in [4]–[7]). Additionally, epigenetic signatures have been reported for different brain regions [8], , for regional brain aging [10], and aging in general [11] further supporting epigenetic studies in patients with neurodegenerative diseases.

Progressive supranuclear palsy (PSP) is a neurodegenerative disease typically characterized by parkinsonism, postural instability, and cognitive impairment [12]. Pathologically, PSP is defined by the accumulation of tau protein in subcortical and cortical regions, (reviewed in Williams and Lees, 2009 [13]), showing substantial overlap with other neurodegenerative diseases characterized by tau accumulation and grouped under the generic name of tauopathies, including approximately one-half of all frontotemporal dementia (FTD) cases and Alzheimer's disease [14]. Both rare [15], [16] and common [17] genetic variation have been shown to mediate risk for tauopathies. The major common variant risk for PSP, a prototypical tauopathy, involves a region surrounding the tau locus [18], but how such genetic variation might mediate risk is not known.

We profiled the methylation status in peripheral blood from patients with two tau-related neurodegenerative conditions, PSP and FTD, using Illumina DNA methylation arrays. We then integrated these methylation data with SNP and gene expression data to identify a mediating role for methylation in genetic risk for PSP. We replicate this finding in independent studies and show that it is conserved in brain, providing the first evidence for a role for DNA methylation in mediating the risk for neurodegenerative dementia.

## Results

### Differential methylation analysis

We first analyzed methylation profiles in 171 patients with FTD (n = 128) and PSP (n = 43) and compared them with 185 subjects with no evidence of dementia or other neurological conditions using Illumina HumanMethylation 450 k arrays (Table S1). Two datasets were generated in two batches, samples were compared within each dataset to condition out a potential batch effect, and the resulting differentially methylated probes (DMPs) were combined (see Methods).

**Figure 1:**
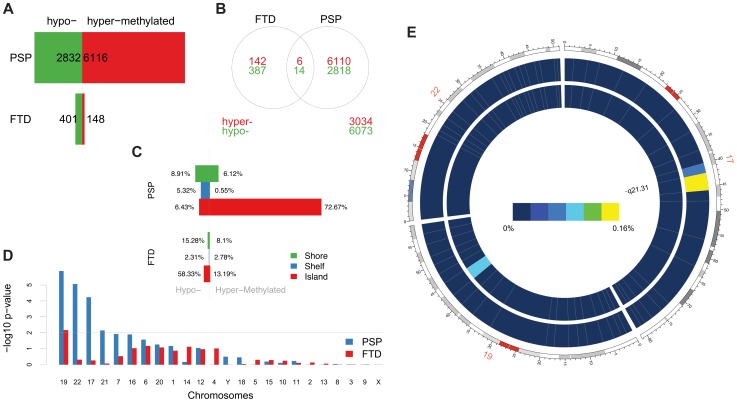
Differentially methylated probes (DMPs) identified in disease vs. control comparisons. (**a**) Barplots representing the numbers of differentially methylated probes (DMPs) identified in each disease group vs. controls (Benjamini-Hochberg-adjusted *p*-value ≤0.05). The number of DMPs indicated in PSP vs. Control comparison is the union set of DMPs identified in dataset #1 and dataset #2. Red bars: hypermethylated DMPs, green bars: hypomethylated DMPs. (**b**) Venn diagram representing the overlap between DMPs in FTD vs. controls and PSP vs. controls. Red numbers: hypermethylated DMPs; green: hypomethylated DMPs. (**c**) Barplots representing DMPs classified by probe type. CpG island probes are overrepresented in both FTD vs. controls and PSP vs. controls. (**d**) Chromosome enrichment analysis: DMPs are significantly enriched in chromosomes 19, 22, and 17, only in PSP vs. controls (y axis: −log10 (p-value), hypergeometric test). (**e**) Circos plot [65] of chromosomes 19, 22, and 17 showing regional enrichment of DMPs (PSP vs. Control comparison, BH adjusted p-value ≤0.05, absolute average beta difference (aβD)>0.1) in one region on chromosome 17. Each chromosome was divided into 20 regions, which contain the equal number of CpG probes. Regions were colored according to the DMP density. Blue: low DMP density, yellow: high density. Circles from inner to outer represent FTD, PSP vs. controls, respectively.

Differential methylation analysis identified a number of DMPs between affected subjects and controls, with partial overlap between PSP and FTD (Figure 1a–b, complete list of DMPs is in Table S2). DMPs were mostly clustered within CpG islands (defined according to the Illumina annotation), with most being hypermethylated in PSP vs. controls (Figure 1c, Table 1). Gene ontology analysis of DMPs in PSP vs. controls showed overrepresentation of genes involved in a number of pathways, including DNA binding and transcription factor binding (Figure S1). We then assessed the chromosomal distribution of the DMPs, and observed – only in PSP samples vs. controls – an overrepresentation of probes from chromosomes 19 (hypergeometric test *p*-value = 1.32×10^−6^), 22 (*p* = 8.63×10^−6^), and 17 (*p* = 5.82×10^−5^, Figure 1d), with most top DMPs (after filtering for absolute average beta difference (aβD)>0.1) located within the 17q21.31 region (Figure 1e). The most significant DMPs when comparing PSP vs. controls (n = 14, absolute aβD>0.1) are listed in Table 2. Of note, 4 DMPs (all hypomethylated in PSP) were located within the *NFYA* gene, encoding for a component of a nuclear transcription factor. Importantly, 3 of the 14 significant DMPs are located in 17q21.31 (Figure 1e, *p* = 2.23×10^−7^, hypergeometric test). Despite being located in a relatively limited genomic region, these 3 probes were both hypermethylated and hypomethylated in PSP vs. controls, suggesting complex disease-associated patterns of differential methylation in this region.

**Table 1:** DMPs identified in disease vs. controls classified by probe type (Island, Shelf, and Shore).

	Methylation status	Island	Shelf	Shore	p-value	overall p-value
**FTD**	Hyper	57	12	35	0.709	2.32×10^−17^
	Hypo	252	10	66	1.18×10^−20^	
**PSP**	Hyper	5413	41	456	0	0
	Hypo	479	396	664	2.12×10^−70^	

Hypomethylated DMPs are overrepresented in CpG islands when comparing FTD vs. controls, and both hyper- and hypomethylated DMPs are overrepresented in CpG islands in PSP vs. controls. Chi-square test was performed within hyper and hypo-methylated DMPs (p-value column) or across all DMPs (overall p-value column) by considering the proportion of all probes on the chip as population frequencies.

**Table 2:** Top DMPs identified in PSP vs. controls, after filtering for an adjusted p-value ≤0.05, and an absolute average beta difference (aβD)≥0.1.

Probe ID	aβD	aMeth	P-value	Adjusted P-value	Gene	Position	Probe Type
cg03865648	−0.105	0.286	3.37×10^−5^	0.007	/	chr3:173113856	N_Shore
cg09580153	−0.100	0.920	4.55×10^−5^	0.008	NFYA	chr6:41068724	Island
cg12000995	−0.109	0.535	5.23×10^−5^	0.009	KRTCAP3	chr2:27665139	Island
cg03644281	−0.105	0.916	8.78×10^−5^	0.012	NFYA	chr6:41068752	Island
cg04346459	−0.131	0.876	9.03×10^−5^	0.012	NFYA	chr6:41068666	Island
cg03428951	−0.133	0.588	1.16×10^−4^	0.014	FAM153C	chr5:177434336	S_Shore
cg25110423	−0.112	0.848	1.23×10^−4^	0.015	NFYA	chr6:41068646	Island
**cg23758822**	**0.105**	**0.205**	**1.74×10^−4^**	**0.018**	**/**	**chr17:41437982**	**N_Shore**
**cg22968622**	**−0.127**	**0.160**	**1.85×10^−4^**	**0.019**	**/**	**chr17:43663579**	**Island**
cg22295435	0.140	0.564	1.95×10^−4^	0.019	VSTM2A	chr7:54615864	S_Shore
cg21819782	−0.133	0.294	3.89×10^−4^	0.028	/	chr2:62609317	NA
**cg12609785**	**−0.103**	**0.129**	**4.10×10^−4^**	**0.029**	**/**	**chr17:43660871**	**N_Shore**
cg24401049	−0.102	0.578	7.21×10^−4^	0.040	ARHGAP6	chrX:11157158	Island
cg12289251	−0.106	0.389	8.08×10^−4^	0.043	CACNB2	chr10:18689471	NA

ID: Illumina probe ID; aβD: average beta difference, aMeth: average Methylation level. In bold are probes located within the 17q21.31 region.

### 17q21.31 haplotype and methylation

The location of several DMPs in the 17q21.31 region was intriguing because the 17q21.31 locus contains an established risk factor for neurodegeneration, first reported in 1997 by Conrad *et al.* [18] for PSP and then confirmed in multiple series (reviewed in Wade-Martins, 2012 [19]). Two main haplotypes (H1 and H2) have been described at this locus. The more common H1 haplotype is over-represented (95% vs. 57%) in PSP vs. normal controls [18]–[20]. The H1/H2 locus spans at least 1.8 Mb and includes multiple genes (>40, many of which are actively transcribed in the brain), notably including *MAPT*, encoding for the microtubule-associated protein tau [19]. Mutations in *MAPT* cause FTD and PSP, and hyperphosphorylated tau accumulation is a hallmark in a number of neurodegenerative conditions, including AD, PSP, FTD and others, collectively named ‘tauopathies’.

Consistent with previous reports, the H1 haplotype was overrepresented in our PSP cohort, with a H1 allelic frequency of 97.1% vs. 80.4% in controls (*p* = 1.86×10^−4^, Fisher's exact test, Table S1), further confirming – even in this relatively small data set – the H1 haplotype as a risk factor for PSP. We hypothesized that the clustering of DMPs in 17q21.31 in PSP cases vs. controls might be related to the H1 haplotype risk factor. To detect an effect of the 17q21.31 haplotype on methylation levels, we compared samples based on their genotype at this region, independent of disease classification. As for previous analyses, we compared samples within datasets to avoid potential batch effects. Genotype distribution across diseases and datasets is reported in Table S3.

**Figure 2:**
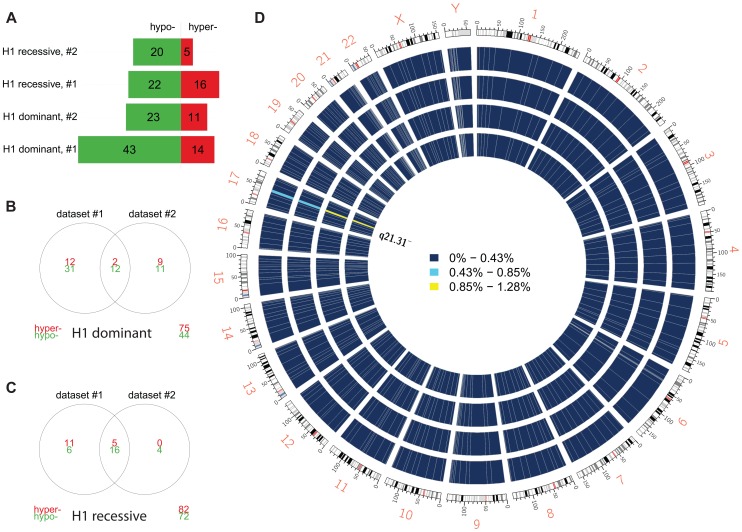
Differential methylation analysis by 17q21.31 haplotype. (**a**) Number of DMPs (Benjamini-Hochberg-adjusted *p*-value ≤0.05) identified in each comparison and each dataset. Dominant: dominant model (H1H1+H1/H2 vs. H2/H2); recessive: recessive model (H1H1 vs. H1/H2+H2/H2). (**b**) Overlap between datasets #1 and #2 (dominant model). (**c**) Overlap between datasets (recessive model). (**d**) Circos plot showing the physical density across the genome of DMPs. Each chromosome was divided in 10 regions, and the proportion of DMPs was assessed. Regions were colored according to the DMP density. Blue: low DMP density, yellow: high density. Circles from inner to outer represent Dataset #2, recessive model; Dataset #1, recessive model; Dataset #2, dominant model; Dataset #1, dominant model. DMPs were mostly enriched in chr17q21.31.

**Figure 3:**
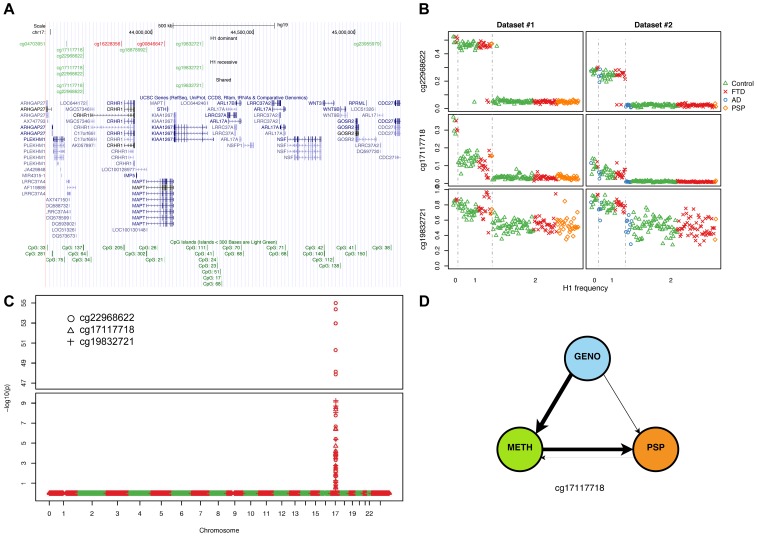
Methylation-QTL at 17q21.31. (**a**) Physical position of top (BH adjusted p-value ≤0.05, absolute average beta difference (aβD)>0.1) DMPs identified when comparing samples based on 17q21.31 haplotype. Dominant: dominant model (H1H1+H1/H2 vs. H2/H2); recessive: recessive model (H1H1 vs. H1/H2+H2/H2); Shared: DMPs shared between the two previous comparisons. Red: hypermethylated, Green: hypomethylated. (**b**) Scatterplot of the methylation levels of 3 top DMPs identified from both H1 dominant and recessive model. (**c**) Methylation-QTL analysis performed in 226 individuals of European descent on 3 the top DMPs identified when comparing H1 vs. H2 haplotypes. Manhattan plot representing p-values by chromosome. At each genomic location the smaller −log10 p-value from two datasets was plotted. A single cluster at 17q21.31 was identified for all three DMPs. (**d**) Results of network edge orienting (NEO) analysis for the differentially methylated probe cg17117718 following the mediation model (GENO causes METH causes PSP). The arrow line thickness is proportional to the likelihood that the edge is oriented in the causal direction, found by calculating the relative probability of the model likelihoods determined by NEO.

We compared carriers of the risk-associated H1 haplotype (H1/H1 and H1/H2 genotypes) to H2/H2 samples (dominant model) within each dataset and, after filtering DMPs for adjusted *p*≤0.05, identified two overlapping sets of 57 and 34 DMPs (Figure 2a,b), markedly clustered within the 17q21.31 region (Figure 2d). Similar results (Figure 2a,c) were obtained when comparing H1/H1 samples to H2 carriers (H1/H2 and H2/H2, recessive model), supporting the hypothesis of a strong *cis* effect of the H1/H2 locus on methylation levels in peripheral blood (Figure 2). After filtering for absolute aβD>0.1, 8 of the top 9 DMPs identified in both datasets were within 17q21.31 (Table 3) in the dominant model. As noted in PSP cases vs. controls, DMPs in this region are both hyper- and hypo-methylated, suggesting a complex cis-regulation of methylation levels (Figure 3a). Scatterplots of the methylation levels for the top DMPs shared between the dominant and recessive models indicate that the H1 haplotype influences methylation levels at these sites in a dose-dependent fashion (Figure 3b), accounting for a majority of methylation variability at these sites (e.g. R-squared = 0.835 and 0.866 in dataset #1 and #2, respectively, for cg22968622). Similar results were obtained when comparing subjects based on their genotype at 17q21.31, but only within controls, FTD, or AD patients (Text S1). The H1 haplotype can be further divided into sub-haplotypes [21]. We obtained sub-haplotype information for 93 H1 carriers in our cohort using the SNPs described in Kauwe et al 2008 [22]. Hierarchical clustering of the methylation signal in the 17q21.31 region and principal component analysis did not reveal a particular clustering of H1 sub-haplotypes (data not shown). These results – although based on a subset of our cohort – suggest that haplotype structure is not the major determinant of 17q21.31 methylation overall.

**Table 3:** DMPs identified when comparing 17q21.31 H1 carriers to non-carriers (dominant model, absolute average beta difference (aβD)>0.1, adjusted p-value ≤0.05).

	Dataset #1		Dataset #2				
ID	aβD	adjusted p	aβD	adjusted p	Gene	Coordinate	Type
cg18878992	−0.185	3.71×10^−55^	−0.341	1.81×10^−86^	MAPT	chr17:43974344	Island
cg17117718	−0.263	7.00×10^−28^	−0.212	3.90×10^−19^	/	chr17:43663208	Island
cg07870213	−0.203	3.50×10^−19^	−0.174	2.82×10^−23^	DND1	chr5:140052090	Island
cg16228356	0.167	8.74×10^−12^	0.145	2.70×10^−04^	/	chr17:43848958	/
cg04703951	−0.186	5.70×10^−08^	−0.158	2.04×10^−07^	/	chr17:43578652	/
cg23955979	−0.215	9.14×10^−06^	−0.185	3.19×10^−12^	/	chr17:45126661	/
cg00846647	0.133	6.95×10^−05^	0.178	6.62×10^−19^	MAPT	chr17:44060252	Island
cg19832721	−0.254	8.59×10^−03^	−0.295	1.73×10^−05^	KIAA1267	chr17:44249866	/
cg22968622	−0.342	2.26×10^−02^	−0.369	2.04×10^−07^	/	chr17:43663579	Island

To test the contribution of haplotype status on PSP-associated DMPs, we repeated the differential methylation analysis only on samples with the H1/H1 haplotype (n = 31 PSP cases and 59 unaffected controls). Of the resulting 341 significant DMPs (after application of the Benjamini-Hochberg procedure, FDR = 0.05), 21 were located in chromosome 17 and 2 were located in the 17q21.31 band. Neither the chromosome nor the region were found to be significantly overrepresented by the hypergeometric test (*p* = 0.342 and 0.149, respectively). The lack of overrepresentation within 17q21.31 after conditioning on strata defined by the 17q21.31 haplotype suggests that either the previously identified overrepresentation on chromosome 17 was due to the 17q21.31 haplotype effect on methylation levels, or that it could also reflect reduced power due to small sample size. To address the issue that the strata contained too few samples, we also carried out a multivariate regression model analysis that included 17q21.31 haplotype as covariate. Specifically, the methylation level of each of the 3 top PSP-related DMPs located in 17q21.31 (Table 2) was regressed on PSP status, 17q21.31 haplotype, ethnicity, and age using a multivariate linear regression model. We found that, except for cg23758822, other PSP-related DMPs were no longer significant (*p* = 0.410 on average, Table S4) in a multivariate model once it included the H1 genotype. We also calculated the relative weight of each predictor using the R package relaimpo [23], and determined that the H1 haplotype accounted for the majority of explained variance (78.2±25.9%, Figure S2). Finally, we estimated relative cell count composition in peripheral blood using methylation data [24]–[26]. Correction for inferred cell count did not significantly change our findings (Text S1, Table S5, Figure S11).

Taken together, these findings indicate 1) a strong effect of the 17q21.31 haplotype on methylation levels at 17q21.31, 2) that the risk-associated H1 determines most of the methylation changes observed with confidence in PSP patients vs. controls, and 3) that additional DMPs outside the 17q21.31 region may be at play in determining risk susceptibility for PSP in H1 carriers, though larger sample sizes will be needed to clarify their importance.

### Genome-wide methylation QTL analysis confirms a *cis* methQTL at 17q21.31

Our findings strongly indicate a *cis* regulation of methylation levels at the 17q21.31 locus. To test whether there were additional potential genetic determinants of methylation levels at 17q21.31 in our dataset, we performed a methylation QTL (methQTL) analysis in a subset of 226 individuals of European descent for whom whole-genome SNP and methylation data were available (Table S6). We assessed association of genetic variants with methylation levels at 3 CpGs within 17q21.31 (cg22968622, cg17117718, cg19832721) in each dataset. We identified on average 110 genome-wide significant signals (Bonferroni-adjusted *p*≤0.05), all located within the 17q21.31 region (Figure 3c, Table S7). These variants accounted for a proportion of variability ranging between 25.5% and 98.2% (mean R-squared = 0.701, Figure S3, Table S8) further confirming that genetic variants at 17q21.31 are controlling methylation levels in *cis* in the same region. We focused on Caucasian individuals because of the differences in frequency of the H2 haplotype across populations. In fact, consistent with previous reports [27], we observed that the H2 haplotype occurs more frequently in Caucasians (H2 allelic frequency = 19.2%, Table 4) than in other ethnic groups (H2 allelic frequency in Asians = 1.3%; *p* = 3.83×10^−6^, Fisher's exact test). However, similar results were observed when including all the 273 individuals for whom SNP and methylation data were available (Text S1, Figure S4, Tables S9, S10).

**Table 4:** Relative distribution of haplotypes at 17q21.31 in ethnic groups.

	Dataset #1			Dataset #2		
	H2H2	H1H2	H1H1	H2H2	H1H2	H1H1
Caucasian	6	42	90	9	33	94
Asian	0	1	18	0	0	21
Latino	1	5	11	2	4	13
Unknown	0	1	4	0	2	2
**Total**	**7**	**49**	**123**	**11**	**39**	**130**

Ethnicity was inferred for 271 samples using SNP clustering compared to Hapmap data (see Methods). Self-reported ethnicity was used for an additional 88 samples.

### *Cis* methQTL effects at the 17q21.31 locus in additional datasets

To confirm that the 17q21.31 haplotype regulates methylation in *cis* at this locus in an independent dataset, we downloaded and reanalyzed raw data from a previously published study, for which SNP and methylation data in peripheral blood from 12 samples were publicly available [28]. Using the rs1052553 SNP to call the H1/H2 haplotype and adopting the same statistical thresholds, we compared H1/H1 vs. H1/H2 subjects and identified one hypomethylated probe (cg22968622, adjusted *p*-value = 2.37×10^−8^, aβD = −0.42) within 17q21.31, which was also identified in our analysis. We also performed a methQTL analysis for cg22968622 in the same dataset. Of the 310 significant SNPs, 206 were located in the 17q21.31 region (*p* = 0, hypergeometric test), further supporting the presence of a *cis* methQTL at this locus.

To provide independent validation of the methylation array assay, we performed reduced representation bisulfite sequencing (RRBS) on a representative set of 7 samples from the study (2 H1/H1 controls, 1 H1/H1 PSP patient, 1 H1/H2 control, 1 H1/H2 PSP patient, and 2 H2/H2 controls). As a sequencing-based approach, RRBS would not suffer from some of the technical biases present in arrays, e.g. due to hybridization. At CpG sites that were covered by both RRBS and array, the methylation measurements were highly correlated (Pearson *r*>0.9) in all seven samples (Figure S5).

To validate our findings from peripheral blood, we analyzed RRBS data from whole-blood DNA of a separate cohort of 80 healthy subjects (comprising 54 H1/H1, 24 H1/H2, and 2 H2/H2). On average, the methylation level computed from RRBS was highly correlated with the array in both dataset #1 (r = 0.965) and dataset #2 (r = 0.963) (Figure S6). Consistent with the array, we found differences in methylation that were significant even after strict Bonferroni correction for multiple testing, mostly localized to the 17q21.31 cytoband (Table S11). Since local methylation levels are often highly correlated, we would expect the differentially methylated CpGs identified by both the array and the sequencing method to be in close proximity. Indeed, the differentially methylated loci identified by RRBS in the 17q21.31 region are nearby those identified by the Illumina Human Methylation array, overlapping the same genes MAPT and KIAA1267. This degree of overlap is striking, given the large extent of the haplotype inversion (Figure S7). Methylated regions identified by the array but not by RRBS may be a result of the higher power (greater sample numbers) in the array, and differences in coverage. Therefore, we looked at probes that both demonstrated haplotype-specific methylation in 17q21.31 on the Illumina array and were covered by RRBS reads in the additional cohort. One probe, cg08113562, met these criteria. The methylation pattern followed a statistically significant dose-dependent relationship with the H1 versus H2 haplotype, with mean methylation fractions of 0.001 in H1/H1 subjects, 0.022 in H1/H2 subjects, and 0.048 in H2/H2 subjects (two-sided p = 0.03, ANOVA), in the same direction as that reported by the array.

To assess the relevance of our findings to brain tissue, we analyzed 2 published methylation QTL studies in brain involving 150 [29] and 153 [30] subjects, respectively. Gibbs *et al.* [29] identified 9 SNP-CpG association pairs (out of 52,345 significant methQTL, mean R-squared = 0.232) at the 17q21.31 locus in two (frontal cortex and cerebellum) of the four studied brain regions; Zhang *et al.* [30] identified in cerebellar samples 122 SNP-CpG pairs (significant in at least one of the three thresholds they used) at the 17q21.31 locus (out of 12,117 significant methQTLs, mean R-squared = 0.136). Together, these data demonstrate that the *cis* methQTL we identified in our study are present in independent studies in peripheral blood, and are preserved in brain.

### Causal inference identifies three methylated regions that may mediate PSP risk

The strong association between the haplotype at 17q21.31 and methylation status raises the question of whether methylation levels mediate the protective or pathogenic effects of haplotype variants. Recent developments in the field of causal inference have yielded quantitative methods to predict the hierarchy of causation given genetic variants [31]–[34]. Network Edge Orienting (NEO), for example, uses structural equation models to choose the best fitting causal model, assuming that the genetic variation is fixed by meiosis and thus “anchors” each model (that is, genotype precedes phenotype) [35]. NEO allows one to evaluate which of five testable causal models (Figure S8) best explains the relationship between genetic variants, methylation levels, and disease status. For instance, the genotype may lead to patterns of methylation that directly contribute to the disease phenotype (Figure S8b). Under this model, the DMPs within 17q21.31 would be the most interesting, as they would correspond to the epigenetic markers mediating the increased risk conferred by the H1 haplotype. Alternatively, the genotype may independently give rise to the methylation and disease phenotype, with neither contributing to the other (Figure S8c). DMPs under this model are only associated with the disease because of the common source of variation due to the 17q21.31 locus.

We applied NEO to calculate a relative fitting index of the “mediation model” for the 9 haplotype-associated DMPs (Table S12) using 35 PSP cases and 184 unaffected controls for whom these data were available. The “mediation model” best explained the methylation pattern in three sites, one of which (cg17117718) was statistically significant (see Methods) (Figure 3d). These results support the hypothesis that methylation status at certain sites likely is a causal mediator of the major known genetic risk related to PSP pathogenesis. Taken together, these results predict – for the first time – a link between epigenetic changes and tauopathies, and will need to be further validated with functional studies.

### Haplotype-associated differences in gene expression

Methylation changes have been associated with changes in gene expression. We examined microarray expression data in peripheral blood available for 120 subjects, to test whether the methylation associated with the 17q21.31 haplotype had such an effect. Among 88 healthy subjects with H1H1 haplotype, 24 healthy subjects with the H1H2 haplotype, and 8 healthy subjects with the H2H2 haplotype, we identified three probes significantly differentially expressed in peripheral blood, mapping to *MAPK8IP1* (on chromosome 11), *LRRC37A4* (located within the 17q21.31 region), and *MTFP1* (on chromosome 22, Benjamini-Hochberg adjusted *p*-values of 2.4×10^−20^, 8.4×10^−5^, and 4.0×10^−2^, respectively). The three probes demonstrated a log fold change of 0.69, −0.25, and 0.10, respectively, in H2 vs. H1 carriers.

The strong haplotype-associated methylation changes identified in our study included DMPs within the *MAPT*, *KIAA1267*, *ARHGAP27*, and *DND1* genes. We used linear regression to test whether the 17q21.31 haplotype was associated with differential expression of these genes, and found no correlation between haplotype and gene expression for these transcripts (adjusted R-squared = 0.006, 0.000, −0.008, and −0.011, respectively). Thus, while haplotype was shown to affect mRNA expression of *MAPK8IP1* and *LRRC37A4* in peripheral blood, there was no detectable correlation between DMP-containing genes and their corresponding expression levels.

## Discussion

The goal of this study was to assess whether changes in DNA methylation in peripheral blood are observed in patients with neurodegenerative diseases. By performing microarray-based differential methylation analysis, we identified a methylation signature associated with disease status in PSP and, to a lesser extent, FTD. Using SNP data available in a subset of our series, we showed that a remarkable proportion of the observed changes in methylation status in PSP are associated with a common haplotype at the 17q21.31 locus, strongly suggesting the presence of a *cis* methylation QTL in this region. Although we included patients with neurodegenerative disorders in our analysis, the observed pattern seems to be related to the haplotype at 17q21.31, independent of disease status. Integrative analyses including SNP and gene expression data support a model whereby genetic variation at the 17q21.31 locus modulates the risk for neurodegenerative tauopathy at least partially via differential methylation.

The H1 haplotype at 17q21.31, a large linkage disequilibrium block due to an inverted chromosomal sequence of ∼970 kb, is the major known risk locus for PSP [36] and other neurodegenerative diseases [17], [37]. Although the genetic contribution of this locus to the risk for neurodegeneration is established and widely replicated, the mechanism by which risk is increased is largely unknown. This region spans from *CRHR1* (corticotrophin) to *IMP5* (a presenilin homologue) at the centromeric end of LD, while *WNT3* and *NSF* (N-ethylmaleimide-sensitive factor) are at the telomeric end of the LD block [38]; therefore it spans at least 1.8 Mb, including 48 RefSeq genes – many of which actively transcribed in the brain – and constitutes the largest haplotype block in the human genome. Stefansson et al. [39] showed that the complete disequilibrium was due to an inversion occurring in the H2 haplotype relative to the H1 human reference and subsequent absence of recombination between inverted and non-inverted chromosomes. The study of this region to understand susceptibility to neurodegeneration has been mostly focused on one gene, *MAPT*, encoding for the microtubule-associated protein tau. This focus on tau is well motivated, as hyperphosphorylated tau accumulates within neurofibrillary tangles – the pathological hallmark of AD – and because mutations in *MAPT* cause FTD, the second most common neurodegenerative dementia. Several *in vitro* studies have reported alterations of transcription levels in *MAPT* due to common variants in the region [40], [41], but this finding has not been consistently replicated [42], [43]. More consistent evidence exists for a higher expression of exon 3 in brains from H2 carriers [43], [44] and of exon 10 in H1 carriers [41], [45], suggesting that splicing abnormalities are involved in increasing risk.

Recently, additional genetic evidence has been reported implicating this locus in other neurodegenerative diseases, such as Parkinson's disease [46], essential tremor, and multisystem atrophy [47]. This is important, as these diseases are not typical tauopathies, suggesting that the effect of this risk-associated region may be complex and involve multiple genes [17].

Our results indicate a novel mechanism by which the H1/H2 locus may affect the risk for tauopathies: significant alterations in methylation mediating increased disease susceptibility. Importantly, these methylation changes are not at the *MAPT* locus only, but are consistently observed in at least 3 neighboring genes as well, suggesting that genes other than *MAPT* might be at play in increasing disease susceptibility. In addition, DMPs in the region were both hyper and hypo-methylated, suggesting a complex regulation of methylation levels at this locus. Further studies will be needed to understand whether the observed methylation signature at 17q21.31 is increasing susceptibility through a *MAPT*-dependent or independent mechanism, or both. Interestingly, a recent study focused on rheumatoid arthritis [48] linked a genetic susceptibility region for the disease, the MHC locus, with methylation changes in the same region, supporting the notion that epigenetic changes might mediate complex disease susceptibility induced by genetic risk factors.

This is the first study of DNA methylation levels in blood in PSP and FTD, disorders that mainly affect brain. Although methylation patterns may be tissue specific [49], comparative studies of blood and brain showed both methylation patterns that are tissue-specific and conserved across tissues [50]. We show that the particular H1 haplotype-related methylation pattern identified in blood is at least partially conserved in brain. This is encouraging, since – in contrast to brain – blood is available from living patients, yielding a higher potential for future use as biomarker and the possibility of large-scale studies. We and others have used gene expression in peripheral blood to gain insights into the biology of neurodegenerative disorders [51], [52]. This study supports the notion that a disease-related signature is present in methylation data as well. Finally, we decided to focus on the risk-associated 17q21.31 region as an initial step, but many interesting candidates for further study emerged from the differential methylation analysis in PSP patients vs. controls, namely the nuclear transcription factor *NFYA*.

Although our analysis of published datasets supports the presence of the 17q21.31-associated methylation signature in brain tissues, further studies focused on brain samples from patients will be needed to test whether methylation changes are contributing to tissue-specific gene expression abnormalities, and ultimately explain the mechanism of action of genetic susceptibility alleles, and the striking regional vulnerability of these disorders.

## Materials and Methods

### Ethics statement

All subjects and/or their proxies signed informed consents for genetic studies. The research protocol was approved by the University of California San Francisco (UCSF) and Los Angeles (UCLA) University Institutional Review Boards for human research.

### Sample description

Patients were enrolled as part of a large genetic study in neurodegenerative dementia (Genetic Investigation in Frontotemporal Dementia, GIFT) at the UCSF Memory and Aging Center (UCSF-MAC) [53]. 371 unrelated subjects were enrolled in the study (Table S1), including patients with neurodegenerative disorders (128 FTD, 43 PSP, and 15 AD), and 185 healthy controls.

### Sample preparation

DNA was extracted from peripheral blood using standard methods. No cell sorting or cell selection was conducted, therefore our data measure methylation levels in whole blood. Total RNA was extracted from the same individuals from peripheral blood using Paxgene Blood RNA tubes (Qiagen).

### Methylation arrays

Whole-genome methylation patterns were assayed by the Infinium Human Methylation450 BeadChip Kit (96 samples per chip). This work was performed in two stages (each including 2 chips), resulting in a total of 371 samples. Samples were hybridized as follows: Dataset #1: PSP (n = 40), FTD (n = 55), Control (n = 93); Dataset #2: FTD (n = 73) AD (n = 15), Control (n = 92).

### Genotyping

#### Taqman genotyping

Genotype variants at APOE (rs429358 and rs7412) and MAPT H1/H2 (rs1560310) were obtained using Taqman assays. Genome-wide SNP data was obtained using the Illumina HumanOmni1-Quad BeadChip. 17q21.31 sub-haplotypes were obtained by genotyping 6 SNPs as previously reported [22] using Taqman assays.

#### SNP arrays

High-throughput SNP genotyping data (Illumina HumanOmni1-Quad BeadChip) from a larger dataset containing 702 samples were available for 273 subjects (14 AD, 110 FTD, 15 PSP, and 134 Controls) in this study. SNP genotypes were called and exported from Illumina GenomeStudio (versions 1.6.3 and 1.8.4). Quality control included filtering for 1) SNPs with <95% genotype call rates (n = 157,121), 2) a minor allele frequency <1% (n = 126,502); and 3) with Hardy-Weinberg Equilibrium *p*-value <1×10^−6^ in the control group (n = 16,954). A total of 788,694 SNPs were included in the final analysis.

#### Ethnicity

We inferred ethnicity for 271 samples (out of the 273 for whom SNP data were available), by using SNP clustering compared to Hapmap data. Briefly, MDS analysis was applied on a merged dataset, including 702 samples from our data (273 of which were included in present study) and 1184 subjects from HapMap phase III. MDS plot shows that the first two principal components can cluster samples by ethnicity, and our data had good overlap with HapMap data (Figure S9). We used self-reported ethnicity for an additional 88 samples. For 12 samples ethnicity remained unknown either because we were not able to call ethnicity with certainty using SNP data (n = 2), or because of the lack of SNP data and self-reported ethnicity (n = 10).

### Microarray-based gene expression analysis

Microarray expression data (Illumina HumanRef-8 v3.0) were available in 120 subjects with H1/H1 or H1/H2 haplotypes at 17q21.31.

### Reduced representation bisulfite sequencing

RRBS was performed on seven samples multiplexed in two lanes of the Illumina HiSeq. Library preparation was performed using the Msp I restriction enzyme as previously described [54]. Read alignment and methylation level calls were performed using BS-Seeker2 [55] (parameters: --aligner = bowtie -m 5 -g hg18.fa -r --low = 50 --up = 500 -a adapter.txt).

### Published datasets

SNP data from Heyn *et al.* [28] reporting the methylome analysis of newborns and centenarians, including 40 samples assayed by Illumina HumanMethylation450 BeadChip and 14 genotyped by Illumina HumanOmni5-Quad BeadChip (both methylation and SNP genotyping data were available for 12 samples), were downloaded from the Gene Expression Omnibus Database (GEO, http://www.ncbi.nlm.nih.gov/geo/, GSE31438). H1/H2 haplotypes were inferred from SNP rs1052553. For methylation data, beta and detection p-values were downloaded from GSE30870, and 65 sites containing missing values were removed.

### Statistical analysis

#### Methylation arrays

In order to avoid potential confounders from batch effects, the two datasets were processed separately. Raw data was processed using the Illumina GenomeStudio software (version 2010.3). Background correction and color normalization were performed using the R package minfi version 1.2.0 [56], and normalization using Subset-quantile Within Array Normalization (SWAN) [57]. Probes were excluded from further analysis if >95% samples had detection p-value >0.01. In summary, 3,027 probes were removed from dataset #1, and 26,306 probes were removed from dataset #2. In order to avoid potential confounders, 66,877 SNP-containing probes were also excluded from further analysis [58]. Beta values (ratio between methylated probe intensity and the overall intensity) were computed using the R package minfi.

Linear models and empirical Bayes methods as implemented in the limma package were used for differential methylation analysis [59]. P-values were adjusted by using the Benjamini-Hochberg (BH) false discovery rate method. Multi-dimensional scaling (MDS) did not show obvious biases between chips within each dataset, but a batch effect could be observed between datasets #1 and #2 (Figure S10), similar to what has been reported in the literature [30], [60]. To avoid potential confounders due to this batch effect, we compared each disease category with the set of controls within the same batch (i.e. conditioning on batch effect). Similar differential methylation results were obtained when we performed a combined analysis across the entire dataset, after correcting for batch effects using ComBat [61] (Text S1). Two filters were applied to conservatively identify differentially methylated sites: 1) a p-value-based filter (BH-adjusted *p*≤0.05) and 2) an absolute average beta difference (aβD) filter (absolute aβD>0.1). Chromosomal enrichment analysis was performed by using the hypergeometric test as implemented in the R phyper function.

The impact of relative cell counts in peripheral blood was estimated as previously described [1], [2] and based on a subset (n = 385) of the 500 loci whose methylation levels reflect the relative proportions of immune cells in unfractionated whole blood. After estimating the blood cell type distribution for each sample using the methylation level of the 385 loci, we applied a linear mixed-effect model considering (1) main blood cell types distribution as dependent variables; (2) disease status (or 17q21.31 haplotype), age, ethnicity, and gender as fixed effects; and (3) chip number as a random effect (Text S1).

Raw and normalized methylation data were deposited in the Gene Expression Omnibus (GEO, www.ncbi.nlm.nih.gov/geo), accession number: GSE53740.

Methylation QTL analysis (methQTL) was performed by regressing methylation level at select CpG sites of interest on SNP genotypes. Age and the first two principal component generated from MDS analysis were also included in the multivariate regression model as covariates. Linear regression methQTL analysis was performed using PLINK [62].

#### Microarray-based expression analysis

Raw data were processed as previously described [63]. Briefly, after quantile normalization, batch effects were removed using ComBat [61]. Differential expression analysis was performed using the limma package [59], applying a false discovery rate filter of ≤0.05 and an absolute log fold change filter of >0.1.

#### Causality analysis

Causality analysis was performed using the software package Network Edge Orienting (NEO) [35], a structural equation modeling software for determining the direction of causality among various phenotypes (e.g. clinical, molecular) given genotype data. Subjects from all batches were included (n = 219). The relative fitting index of the model is estimated by the Single Marker LEO.NB score, defined as the base-10 logarithm of the probability ratio between the mediation model and the next most likely causal model (i.e., a LEO.NB score of 1 means that the fit of the mediation model is 10 times better than that of the next best alternative causal model). The genotype was encoded as the dosage of the minor allele (A) at rs1560310 tagging H2 (i.e., 0 for H1/H1, 1 for H1/H2, and 2 for H2/H2). Gene expression levels were included for differentially expressed probes according to the 17q21.31 haplotype, and were encoded with the ComBat-corrected relative expression levels. Clinical phenotype was encoded as a binary variable (i.e., 0 for unaffected, 1 for affected with PSP). The Single-Marker analysis option was used, and results surpassing the thresholds of a LEO.NB score (a likelihood ratio of model fit) >0.8 and RMSEA index <0.05 were considered significant fits to the mediation model [64].

## Supporting Information

Figure S1 Over-represented gene ontology (GO), molecular function, level 3 (MF_3) categories among DMPs in PSP versus controls (in green the proportion of hypomethylated DMPs; in red the proportion of hypermethylated DMPs) sorted by −log10 (p-value). A −log (p-value) of 1.3 corresponds to an over-representation *p*-value of 0.05.
(PDF)

Figure S2 Relative effect of covariates on methylation beta value variance. The H1 haplotype accounts for most of the explained variance. For the top 3 PSP-related DMPs, the relative importance of predictors in the multivariate linear regression model (including H1 frequency, diagnosis status, age, and ethnicity) was calculated using R package relaimpo. Error bars: 95% bootstrap confidence intervals.
(PDF)

Figure S3 Methylation QTL analysis in 226 individuals of European descent. (**a**) scatterplot representing the R-squared for each of the SNPs in the 17q21.31 region associated with at least one of the three DMPs. Gray: not significant SNPs. (**b**) corresponding genomic region at 17q21.31 (UCSC Genome Browser, hg19). Top significant SNPs controlling the three DMPs are highlighted in red. Age and the first two principal components generated from MDS analysis were added as covariates.
(PDF)

Figure S4 Methylation QTL analysis on the entire dataset (n = 273), performed on 3 top DMPs identified when comparing H1 vs. H2 haplotypes. (**a**) scatterplot representing the R-squared for each of the SNPs in the 17q21.31 region associated with at least one of the three DMPs. Gray: not significant SNPs. (**b**) Manhattan plot representing p-values by chromosome. At each genomic location the smaller −log10 p-value from two datasets was plotted. A single cluster at 17q21.31 was identified for all three DMPs.
(PDF)

Figure S5 Correlation between average methylation fraction (β) values at common CpGs covered by both the Illumina HumanMethylation 450 k BeadChip Array and reduced representation bisulfite sequencing (RRBS), in seven samples from the study. The light blue points represent CpGs within the 17q21.31 cytoband.
(PDF)

Figure S6 Correlation between average methylation fraction (β) values at common CpGs covered by both the Illumina HumanMethylation 450 k BeadChip Array and reduced representation bisulfite sequencing (RRBS), in two independent cohorts. The light blue points represent CpGs within the 17q21.31 cytoband.
(PDF)

Figure S7 UCSC Genome Browser graphic and ideogram for the 17q21.31 inversion region, and 1 Mb of flanking sequencing on each side (which is also in linkage disequilibrium). Differentially methylated regions are depicted above the gene diagrams (blue: identified by Illumina HumanMethylation 450 k Array, and labeled with the Illumina Probe ID number; orange: identified by reduced representation bisulfite sequencing in an independent sample).
(PDF)

Figure S8 Causal models that explain the association between haplotype (HAPL), differentially methylated sites (METH), and PSP status (PSP). (**a**) Overview of the edges that are oriented by Network Edge Orienting (NEO) subroutine. The haplotype is anchored at the beginning of the causal diagram, as genotype precedes methylation and disease temporally (and thus, causally). NEO determines the most likely orientation of the remaining edges for each methylated region. (**b**) The “mediation model,” in which the haplotype-associated effect is mediated by the intermediate step of methylation of a particular site. (**c**) An alternative model, in which the haplotype causes differential patterns of methylation independently from conferring disease risk. (**d–f**) The remaining three alternative causal models considered by NEO.
(PDF)

Figure S9 MDS plot representing the clustering of overlap between the SNP data in 273 samples from this study and Hapmap data. Samples are coded based on self-reported ethnicity.
(PDF)

Figure S10 Multidimensional scaling plot of Illumina 450 K methylation data showing a batch effect between two datasets. No obvious batch effect was observed within each dataset. SNP-containing probes, low-quality probes filtered out in each dataset, and sex chromosome probes were excluded from the analysis. The R function cmdscale was used for MDS analysis.
(PDF)

Figure S11 Volcano plots representing DMPs before and after cell type adjustment, in AD (a), PSP (b), FTD in dataset #1 (c), and FTD in dataset #2 (d).
(PDF)

Table S1 Demographic characteristics of the subjects enrolled in the study.
(DOCX)

Table S2 DMPs identified in each comparison (BH adjusted p≤0.05) in at least one comparison. Red: hypermethylated; Green: hypomethylated.
(XLSX)

Table S3 Breakdown of subjects by disease, and by H1/H2 genotype at the 17q21.31 locus.
(DOCX)

Table S4 R-squared coefficients from multivariate linear regression model for 3 the top PSP-related DMPs.
(DOCX)

Table S5 Significant p-value computed using double-bootstrap standard error. 1000 bootstrap iterations were used for each of the two bootstrap methods of standard error estimation.
(DOCX)

Table S6 Breakdown of the 273 samples for which SNP array data and methylation data are available.
(DOCX)

Table S7 Methylation-QTL analysis for the 3 top DMPs, performed in the 2 datasets separately, in 226 individuals of European descent. Only significant (Bonferroni-adjusted *p*≤0.05) SNPs are presented.
(XLSX)

Table S8 Methylation QTL analysis for 3 DMPs within 17q21.31 in in 226 individuals of European descent.
(DOCX)

Table S9 Methylation-QTL analysis for the 3 top DMPs, performed in the 2 datasets separately, in 273 individuals. Only significant (Bonferroni-adjusted *p*≤0.05) SNPs are presented.
(XLSX)

Table S10 Methylation QTL analysis for 3 DMPs within 17q21.31 in 273 individuals.
(DOCX)

Table S11 Differentially methylated CpGs by genotype, found by reduced representation bisulfite sequencing.
(DOCX)

Table S12 NEO predictions for each identified haplotype-dependent DMP.
(DOCX)

Text S1 Additional analyses including: 1) Testing the 17q21.31 haplotype effect in patients and controls separately; 2) Impact of estimated relative cell counts in peripheral blood; 3) Genome-wide methylation QTL analysis in the entire dataset (n = 273); 4) Differential methylation analysis using the combined dataset (n = 371 samples).
(DOCX)
