# Supplementary figures and images for: An Epigenetic Signature in Peripheral Blood Associated with the Haplotype on 17q21.31, a Risk Factor for Neurodegenerative Tauopathy
Source: PLoS Genet. 2014 Mar 6;10(3):e1004211. doi: 10.1371/journal.pgen.1004211 (PMC3945475; doi:10.1371/journal.pgen.1004211)

# GOTERM\_MF\_3

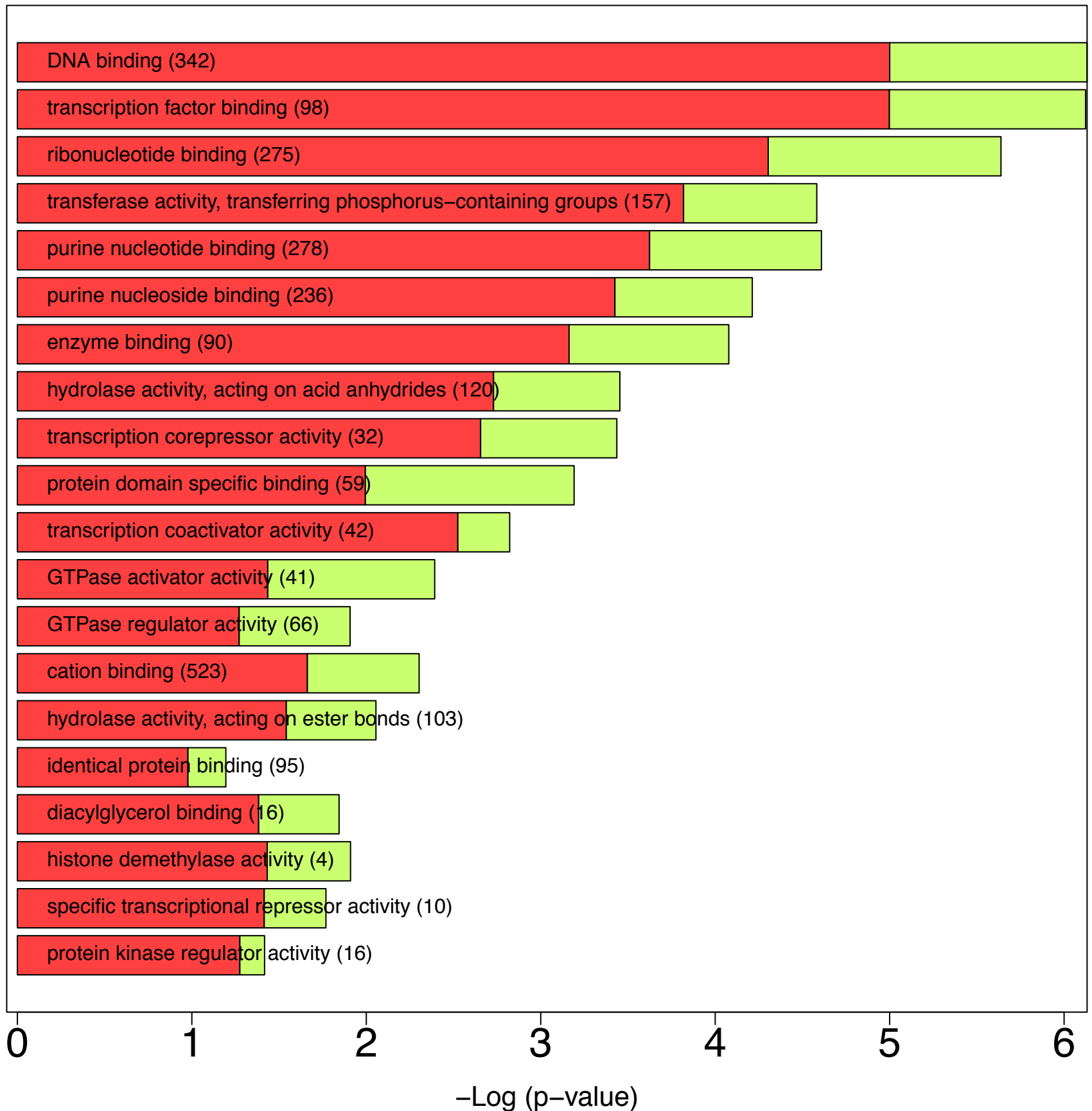

Supplement: Figure S1 — Over-represented gene ontology (GO), molecular function, level 3 (MF_3) categories among DMPs in PSP versus controls (in green the proportion of hypomethylated DMPs; in red the proportion of hypermethylated DMPs) sorted by −log10 (p-value). A −log (p-value) of 1.3 corresponds to an over-representation p-value of 0.05. (PDF) [file pgen.1004211.s001.pdf]

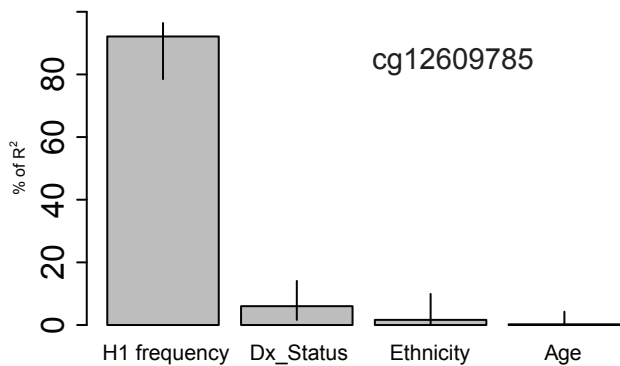

$R^2 = 62.35\%$ , metrics are normalized to sum 100%.

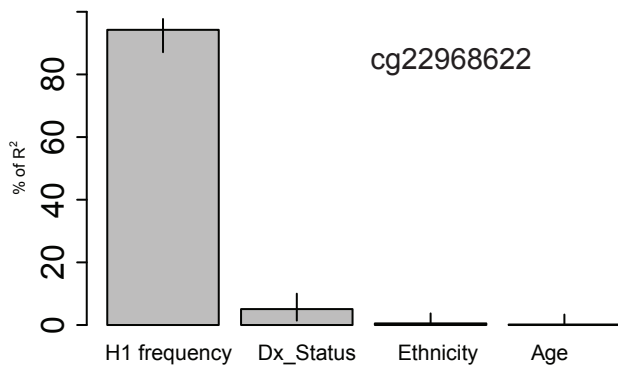

$R^2 = 85.5\%$ , metrics are normalized to sum 100%.

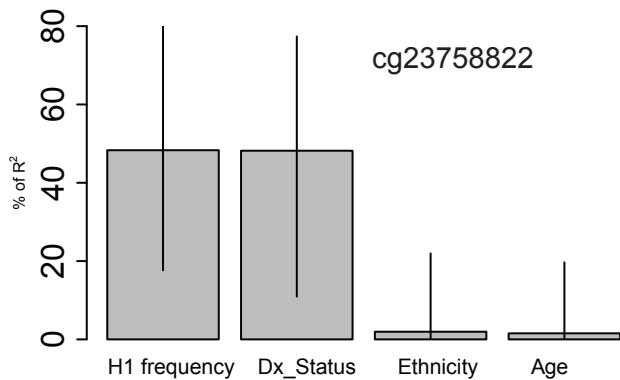

$R^2 = 16.56\%$ , metrics are normalized to sum 100%.

Supplement: Figure S2 — Relative effect of covariates on methylation beta value variance. The H1 haplotype accounts for most of the explained variance. For the top 3 PSP-related DMPs, the relative importance of predictors in the multivariate linear regression model (including H1 frequency, diagnosis status, age, and ethnicity) was calculated using R package relaimpo. Error bars: 95% bootstrap confidence intervals. (PDF) [file pgen.1004211.s002.pdf]

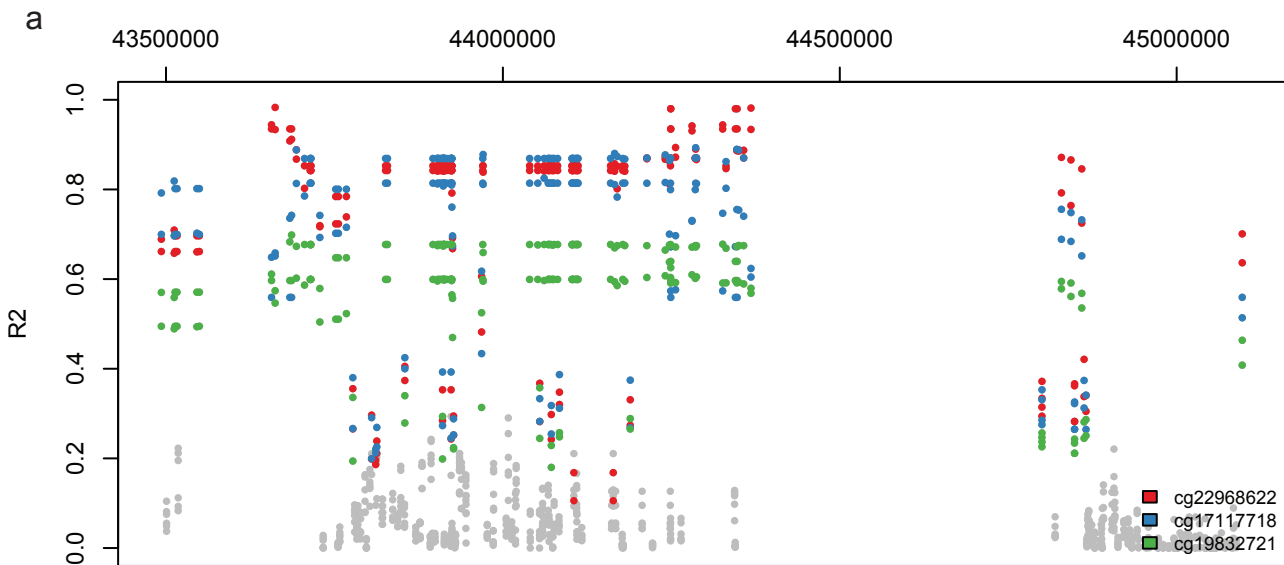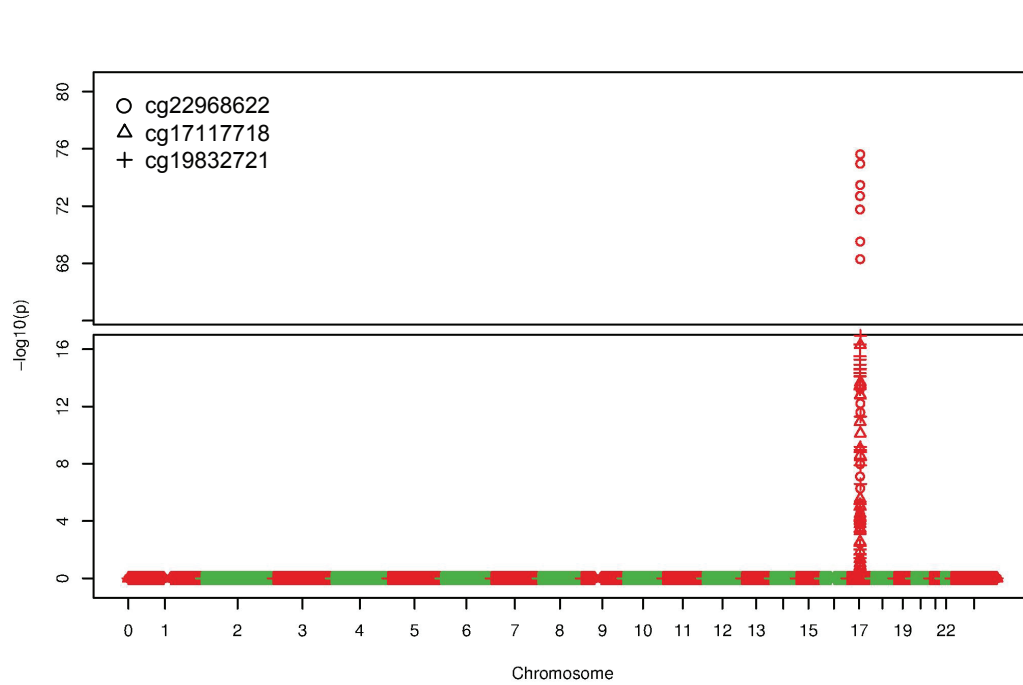

Supplement: Figure S4 — Methylation QTL analysis on the entire dataset (n = 273), performed on 3 top DMPs identified when comparing H1 vs. H2 haplotypes. (a) scatterplot representing the R-squared for each of the SNPs in the 17q21.31 region associated with at least one of the three DMPs. Gray: not significant SNPs. (b) Manhattan plot representing p-values by chromosome. At each genomic location the smaller −log10 p-value from two datasets was plotted. A single cluster at 17q21.31 was identified for all three DMPs. (PDF) [file pgen.1004211.s004.pdf]

1 – H2/H2 Control

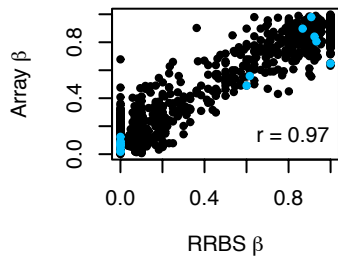

2 – H1/H2 Control

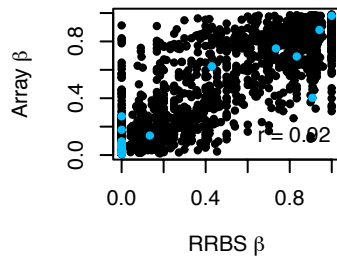

3 – H1/H1 PSP

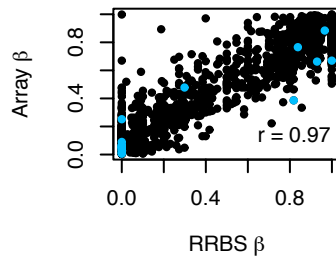

4 – H1/H2 PSP

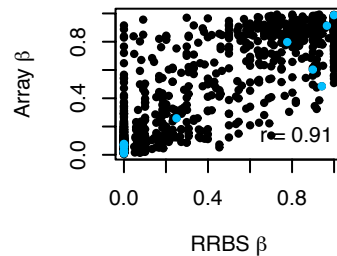

5 – H1/H1 Control

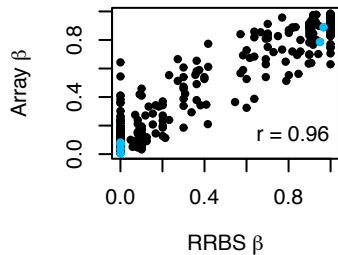

6 – H1/H1 Control

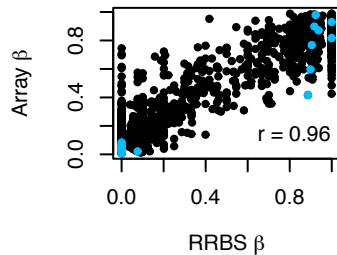

7 – H2/H2 Control

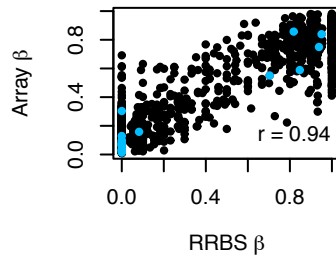

Supplement: Figure S5 — Correlation between average methylation fraction (β) values at common CpGs covered by both the Illumina HumanMethylation 450 k BeadChip Array and reduced representation bisulfite sequencing (RRBS), in seven samples from the study. The light blue points represent CpGs within the 17q21.31 cytoband. (PDF) [file pgen.1004211.s005.pdf]

Illumina HumanMethylation 450k  $\beta$

**Dataset 1**

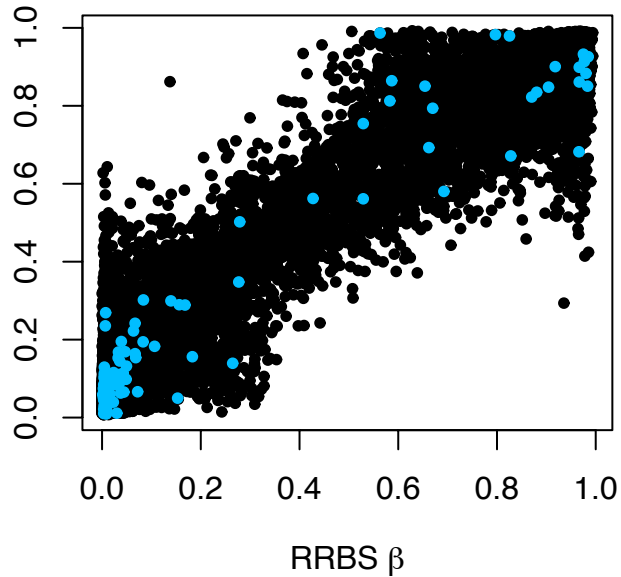

Illumina HumanMethylation 450k  $\beta$

**Dataset 2**

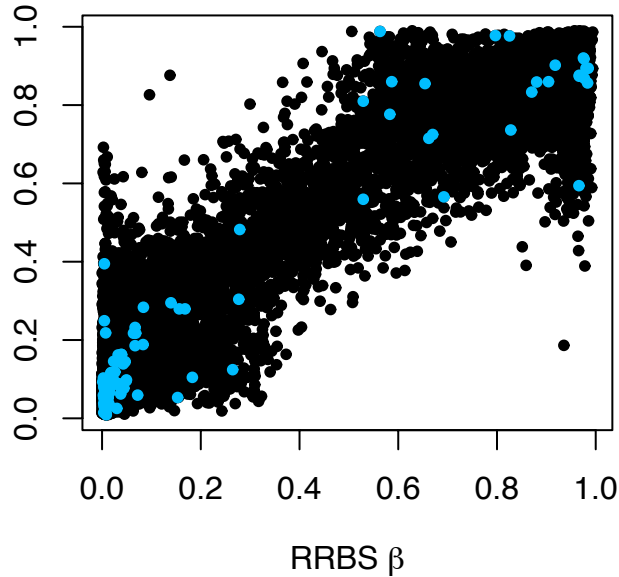

Supplement: Figure S6 — Correlation between average methylation fraction (β) values at common CpGs covered by both the Illumina HumanMethylation 450 k BeadChip Array and reduced representation bisulfite sequencing (RRBS), in two independent cohorts. The light blue points represent CpGs within the 17q21.31 cytoband. (PDF) [file pgen.1004211.s006.pdf]

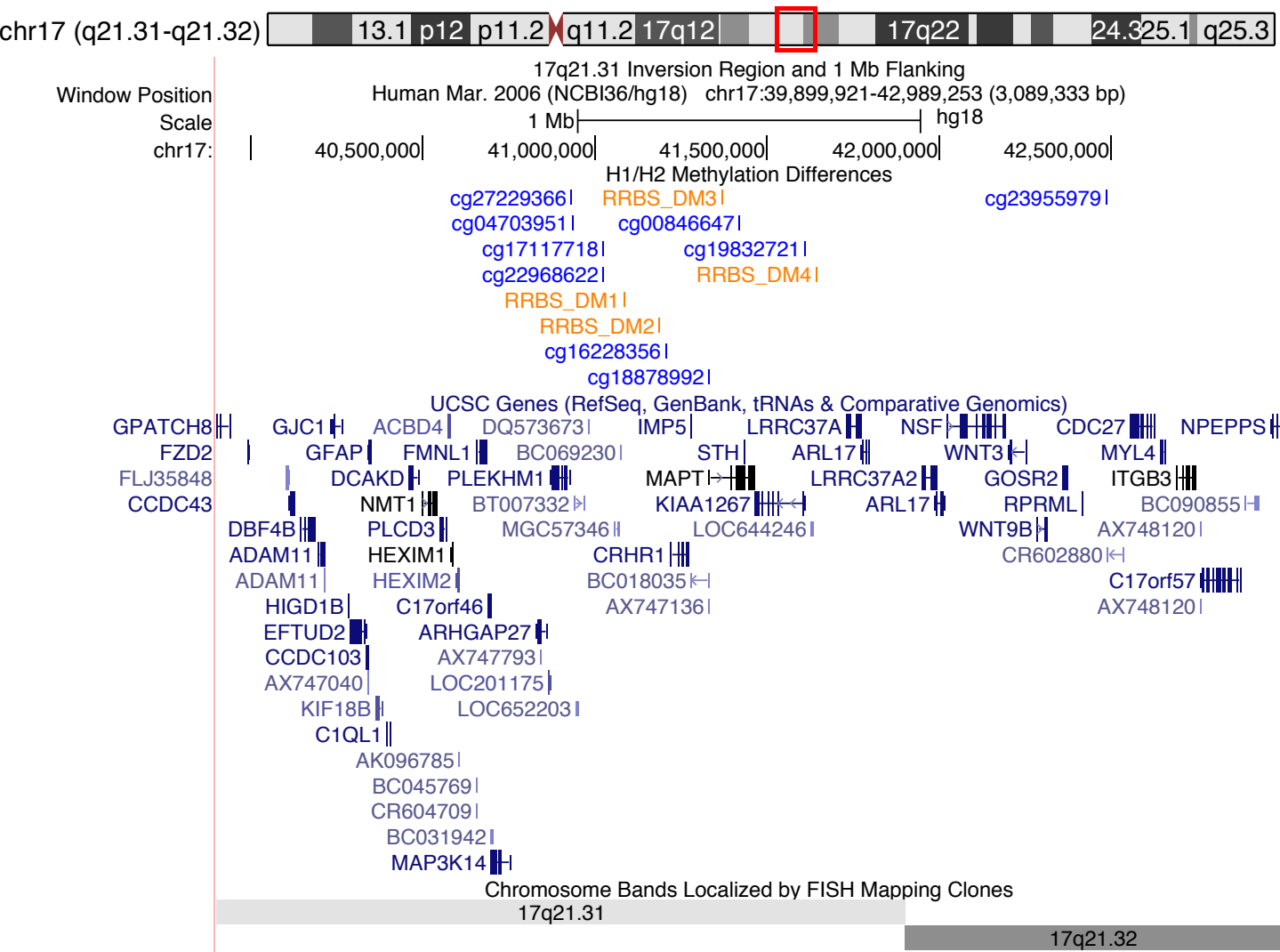

Supplement: Figure S7 — UCSC Genome Browser graphic and ideogram for the 17q21.31 inversion region, and 1 Mb of flanking sequencing on each side (which is also in linkage disequilibrium). Differentially methylated regions are depicted above the gene diagrams (blue: identified by Illumina HumanMethylation 450 k Array, and labeled with the Illumina Probe ID number; orange: identified by reduced representation bisulfite sequencing in an independent sample). (PDF) [file pgen.1004211.s007.pdf]

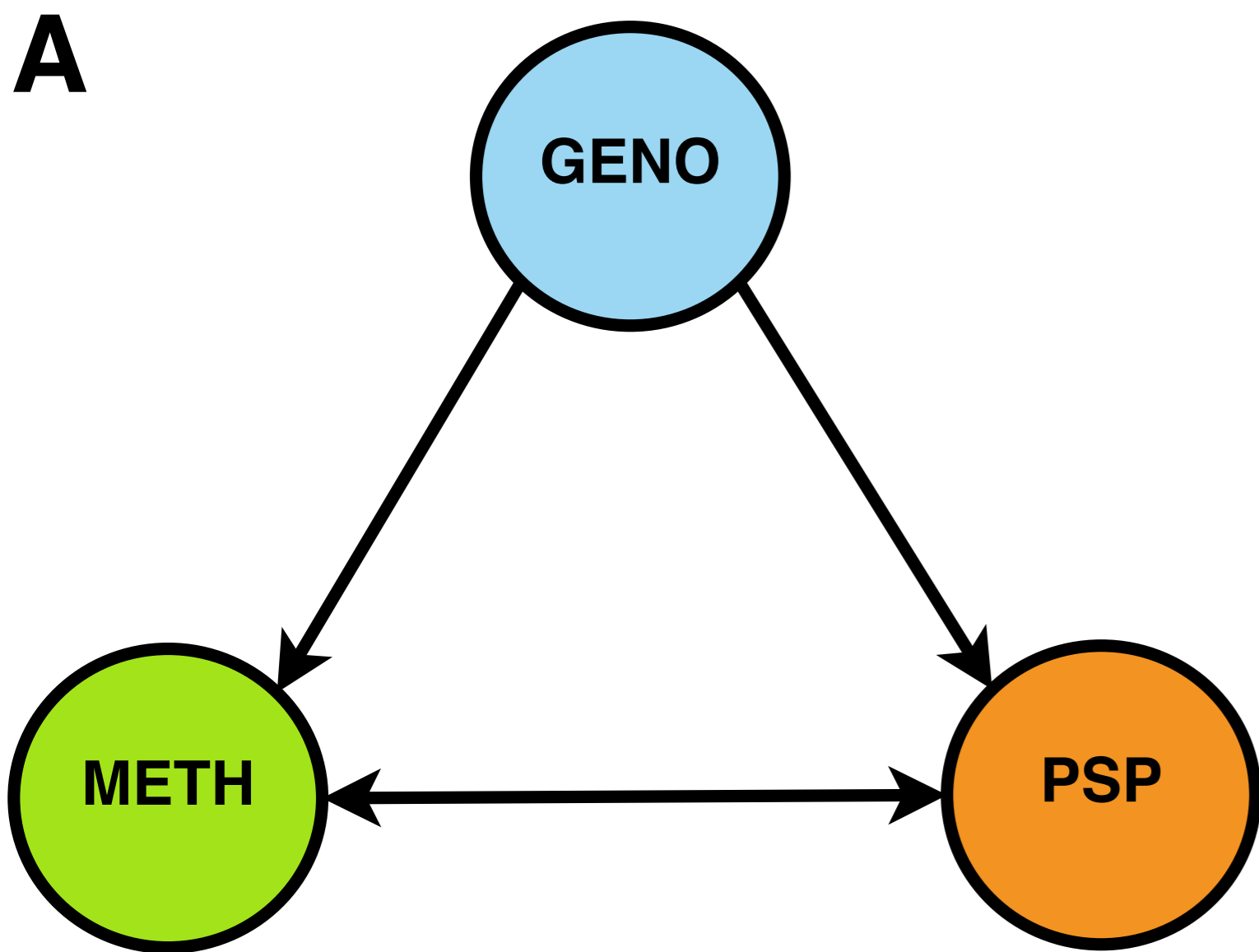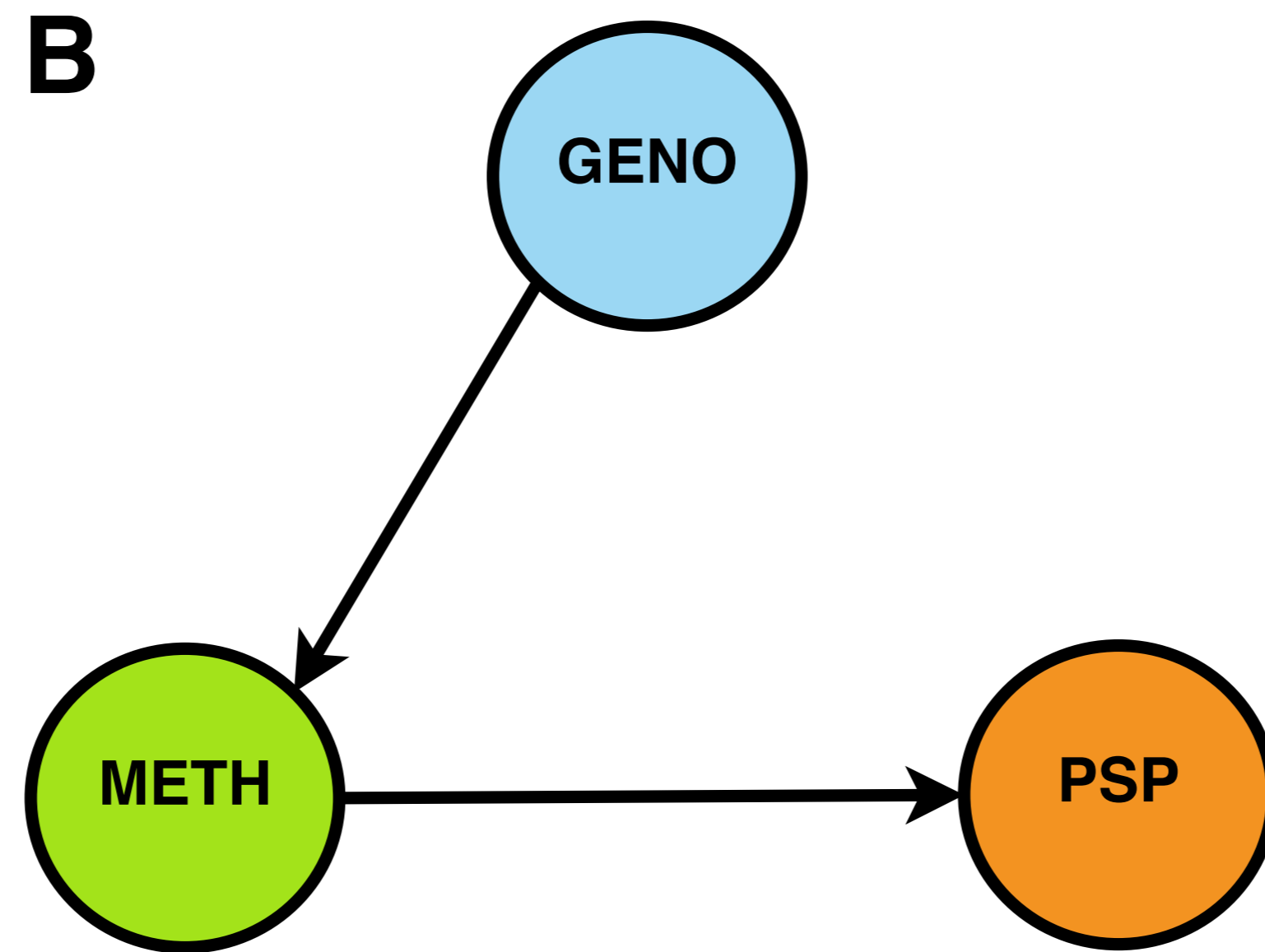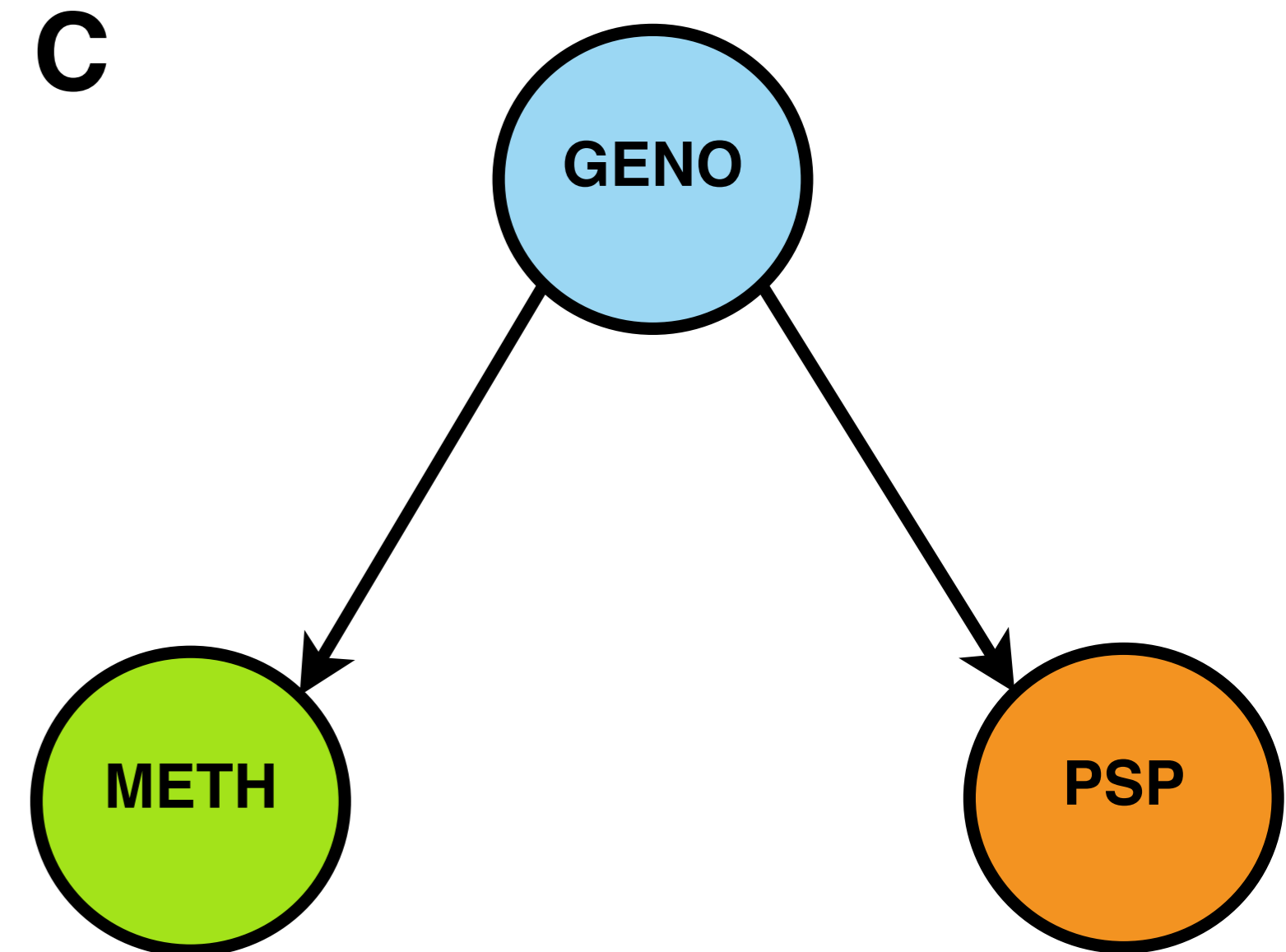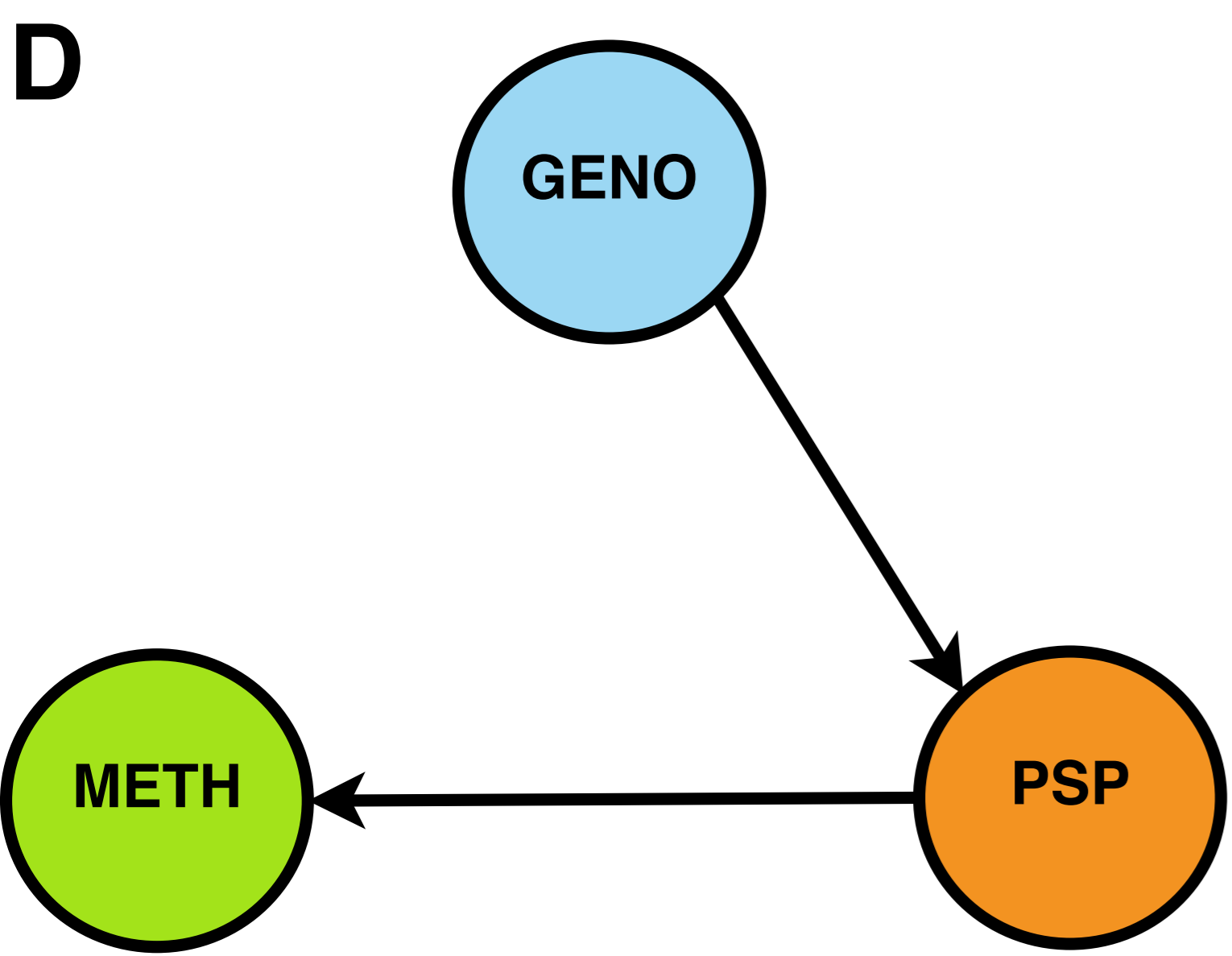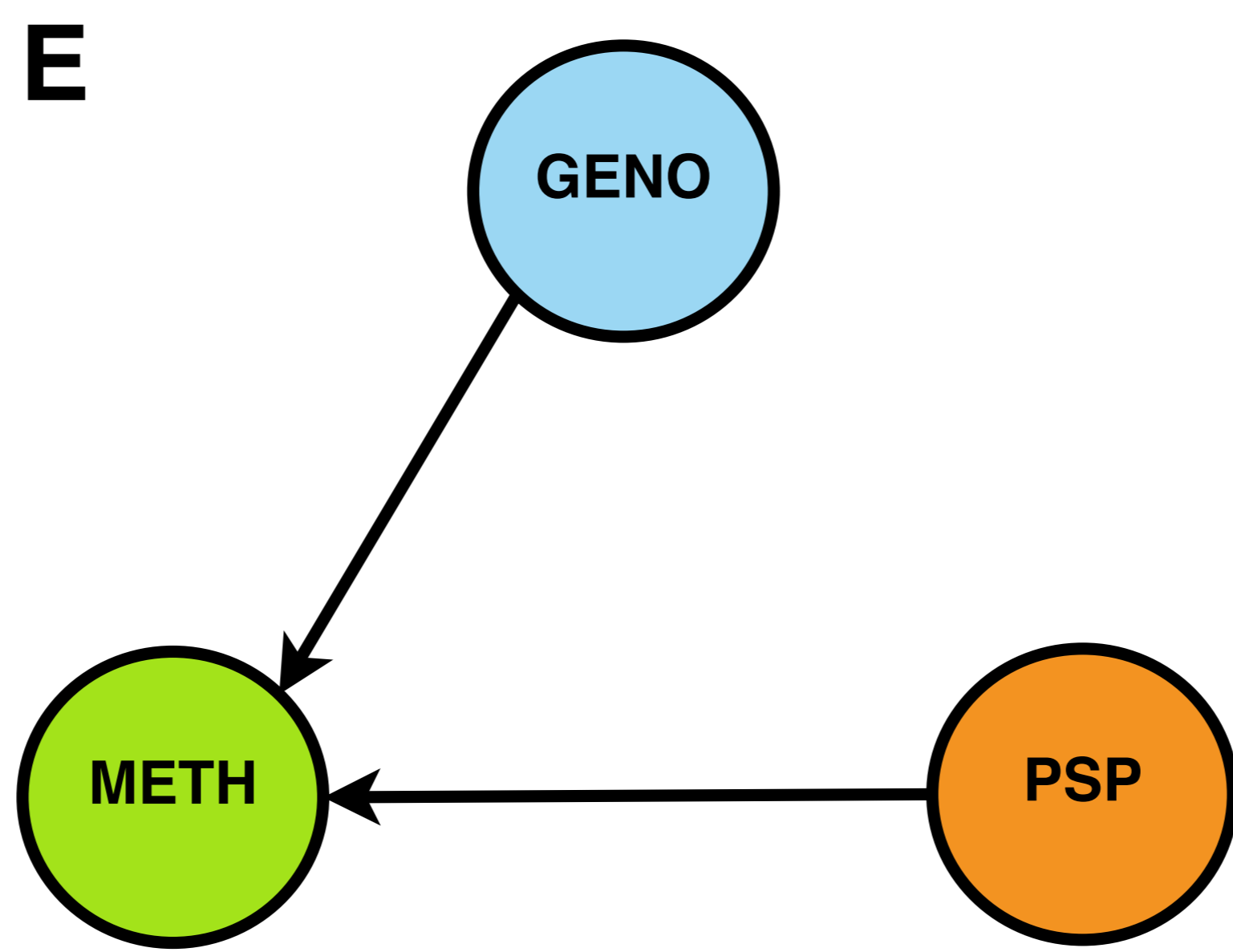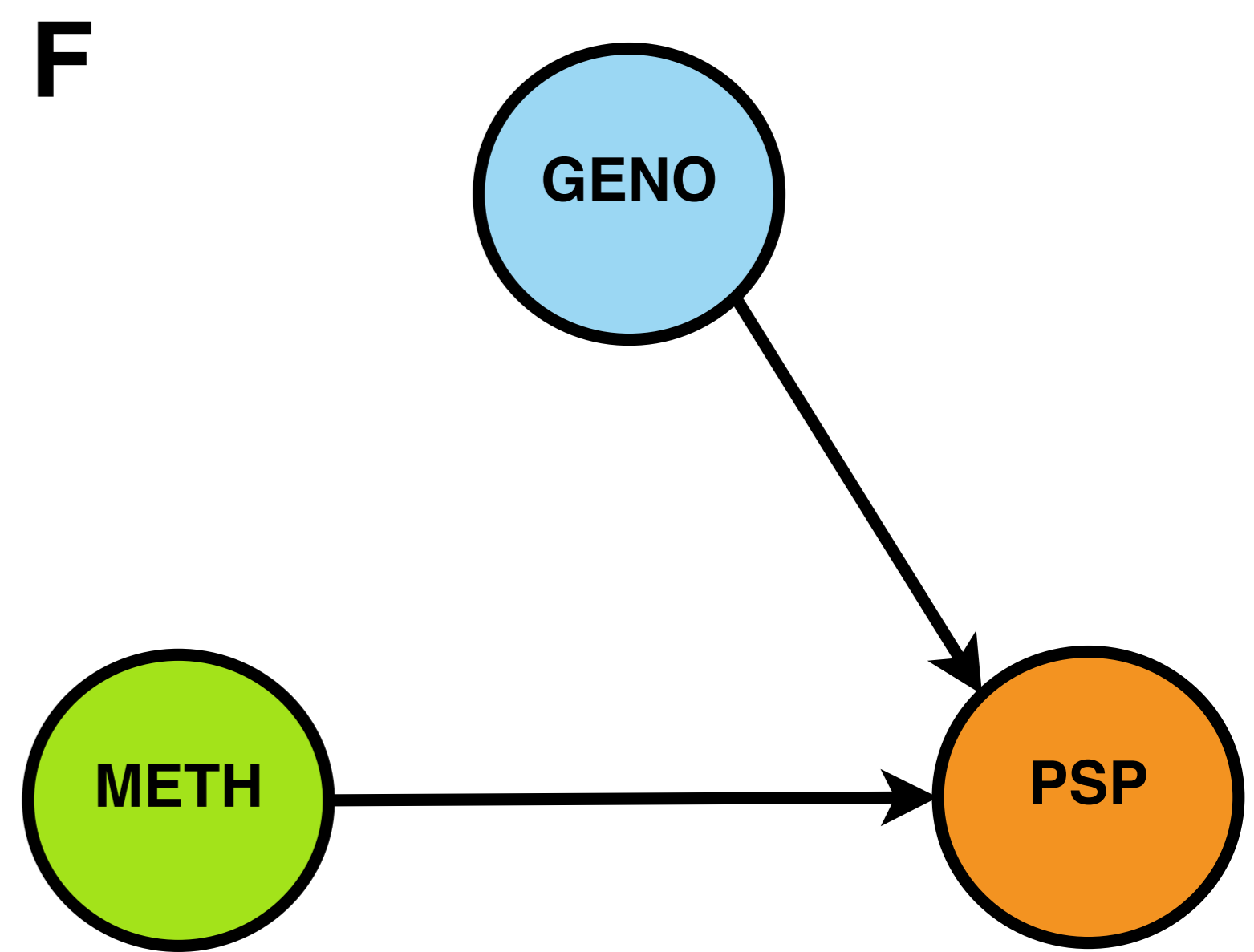

Supplement: Figure S8 — Causal models that explain the association between haplotype (HAPL), differentially methylated sites (METH), and PSP status (PSP). (a) Overview of the edges that are oriented by Network Edge Orienting (NEO) subroutine. The haplotype is anchored at the beginning of the causal diagram, as genotype precedes methylation and disease temporally (and thus, causally). NEO determines the most likely orientation of the remaining edges for each methylated region. (b) The “mediation model,” in which the haplotype-associated effect is mediated by the intermediate step of methylation of a particular site. (c) An alternative model, in which the haplotype causes differential patterns of methylation independently from conferring disease risk. (d–f) The remaining three alternative causal models considered by NEO. (PDF) [file pgen.1004211.s008.pdf]

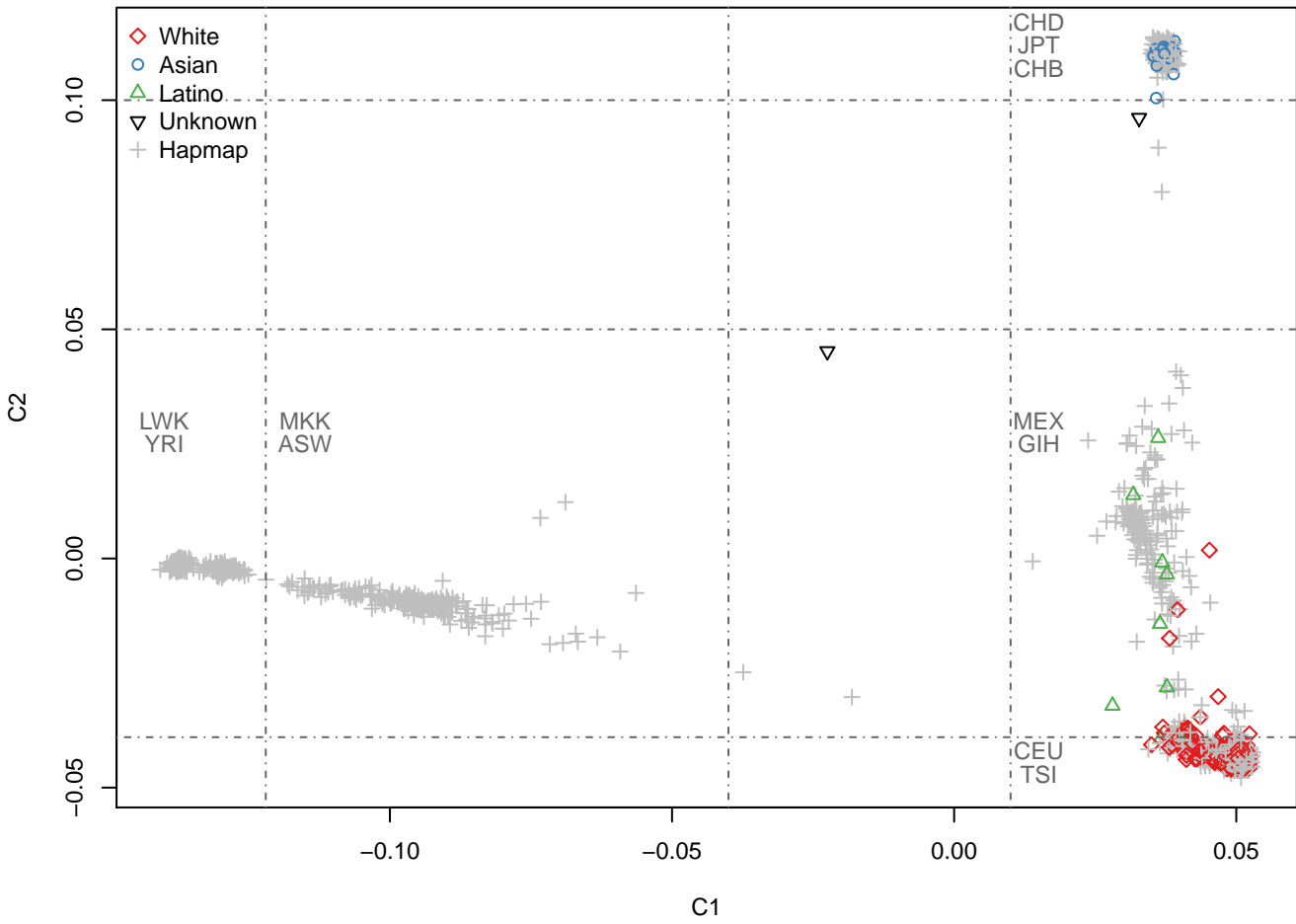

Supplement: Figure S9 — MDS plot representing the clustering of overlap between the SNP data in 273 samples from this study and Hapmap data. Samples are coded based on self-reported ethnicity. (PDF) [file pgen.1004211.s009.pdf]

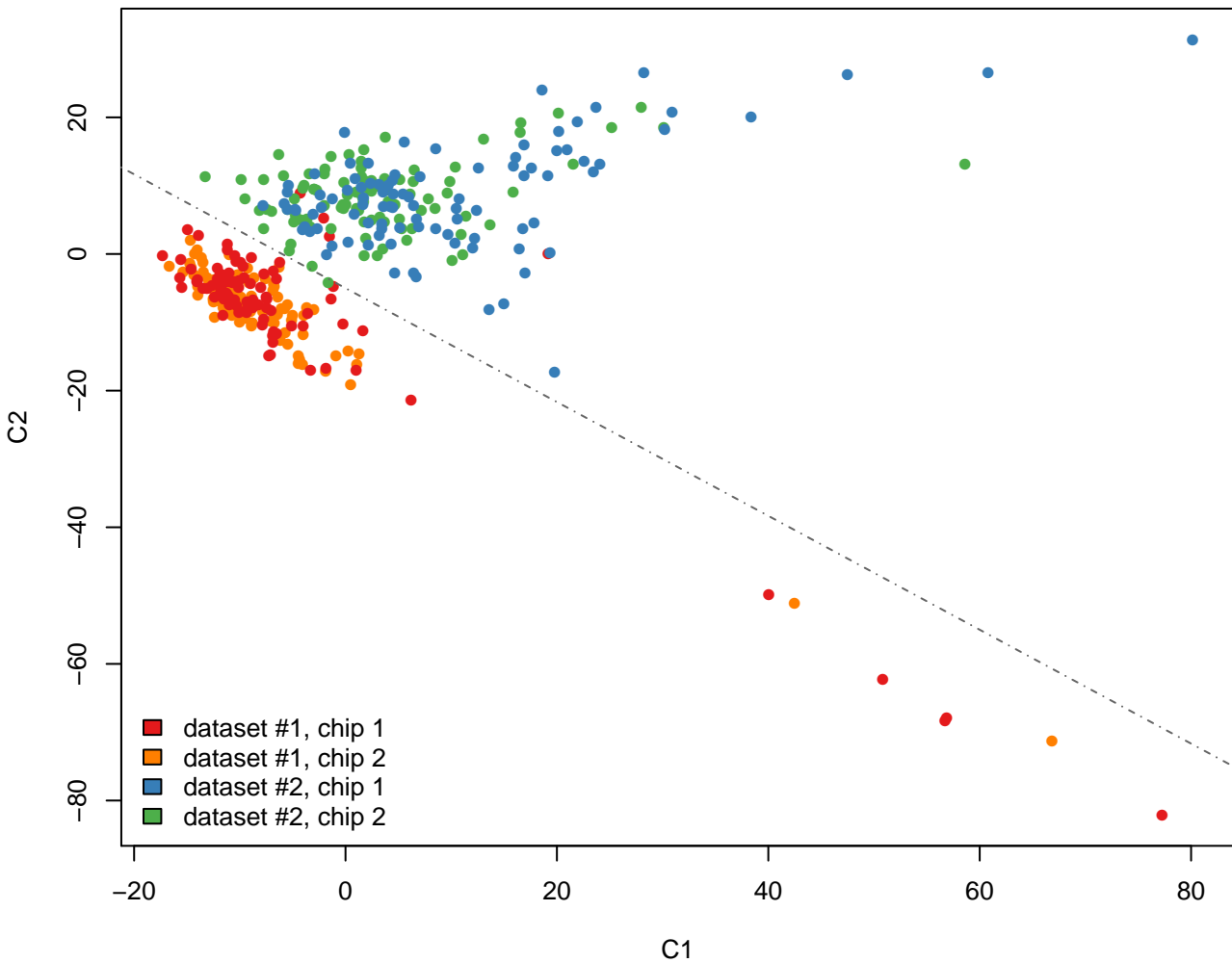

Supplement: Figure S10 — Multidimensional scaling plot of Illumina 450 K methylation data showing a batch effect between two datasets. No obvious batch effect was observed within each dataset. SNP-containing probes, low-quality probes filtered out in each dataset, and sex chromosome probes were excluded from the analysis. The R function cmdscale was used for MDS analysis. (PDF) [file pgen.1004211.s010.pdf]

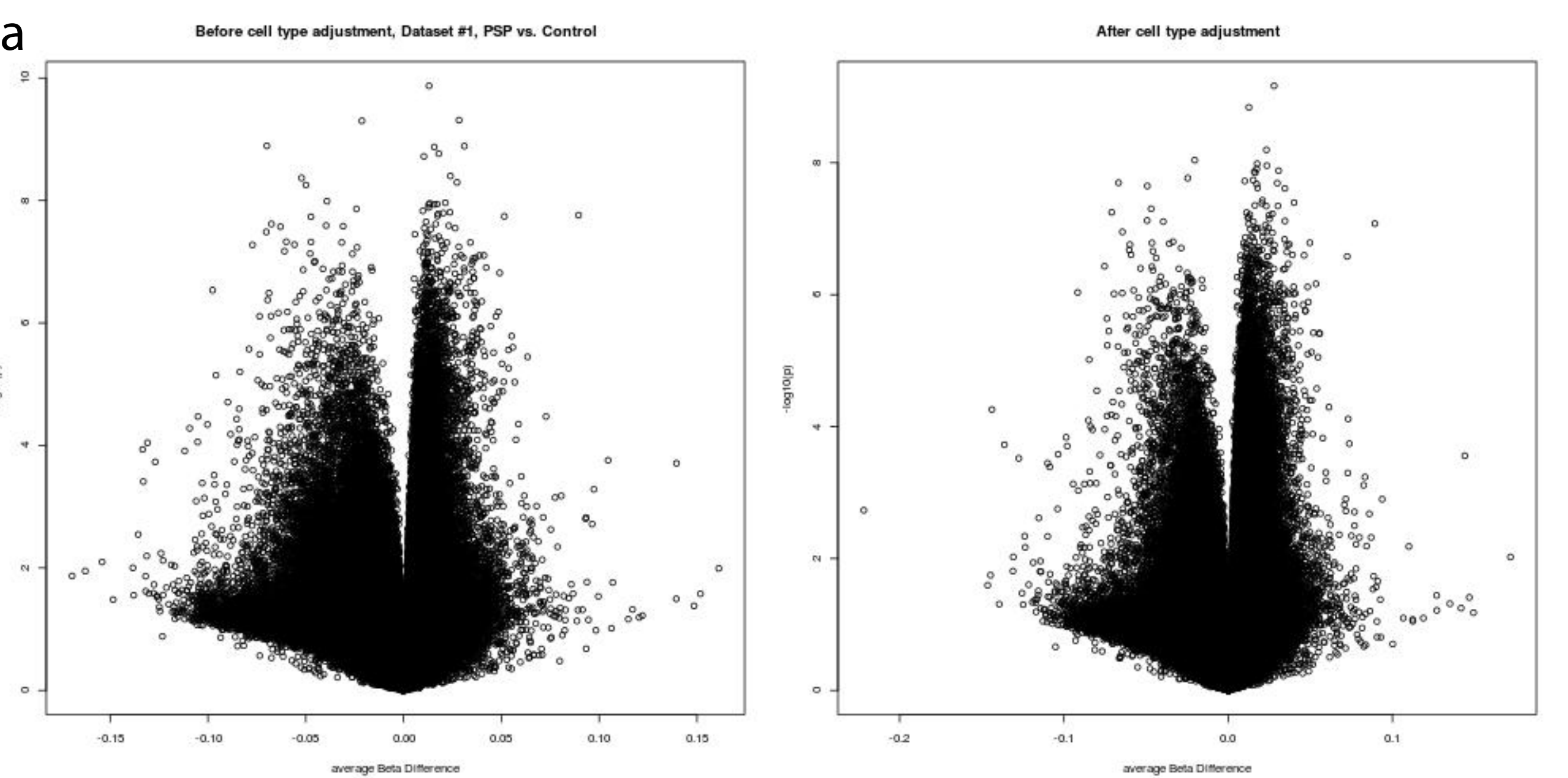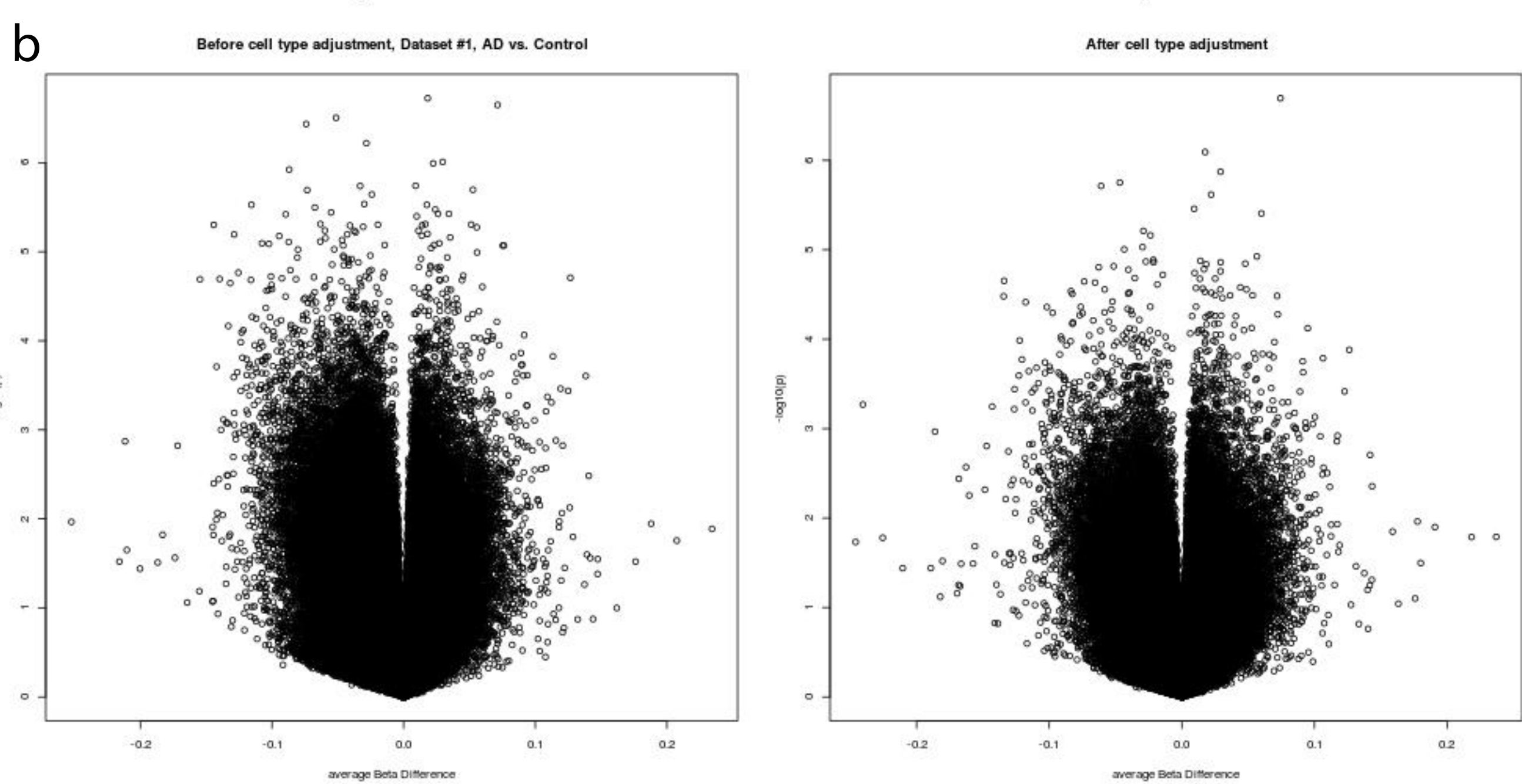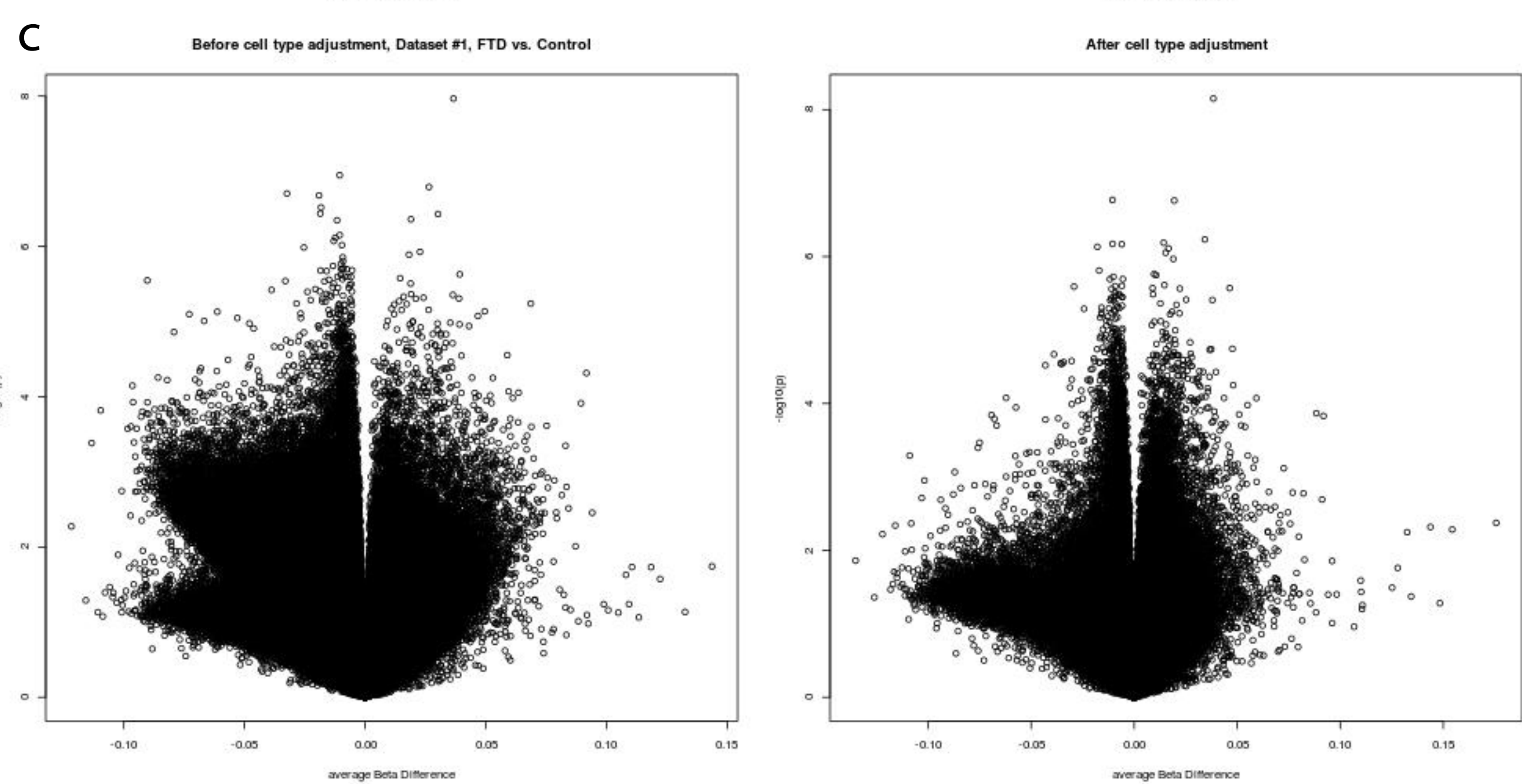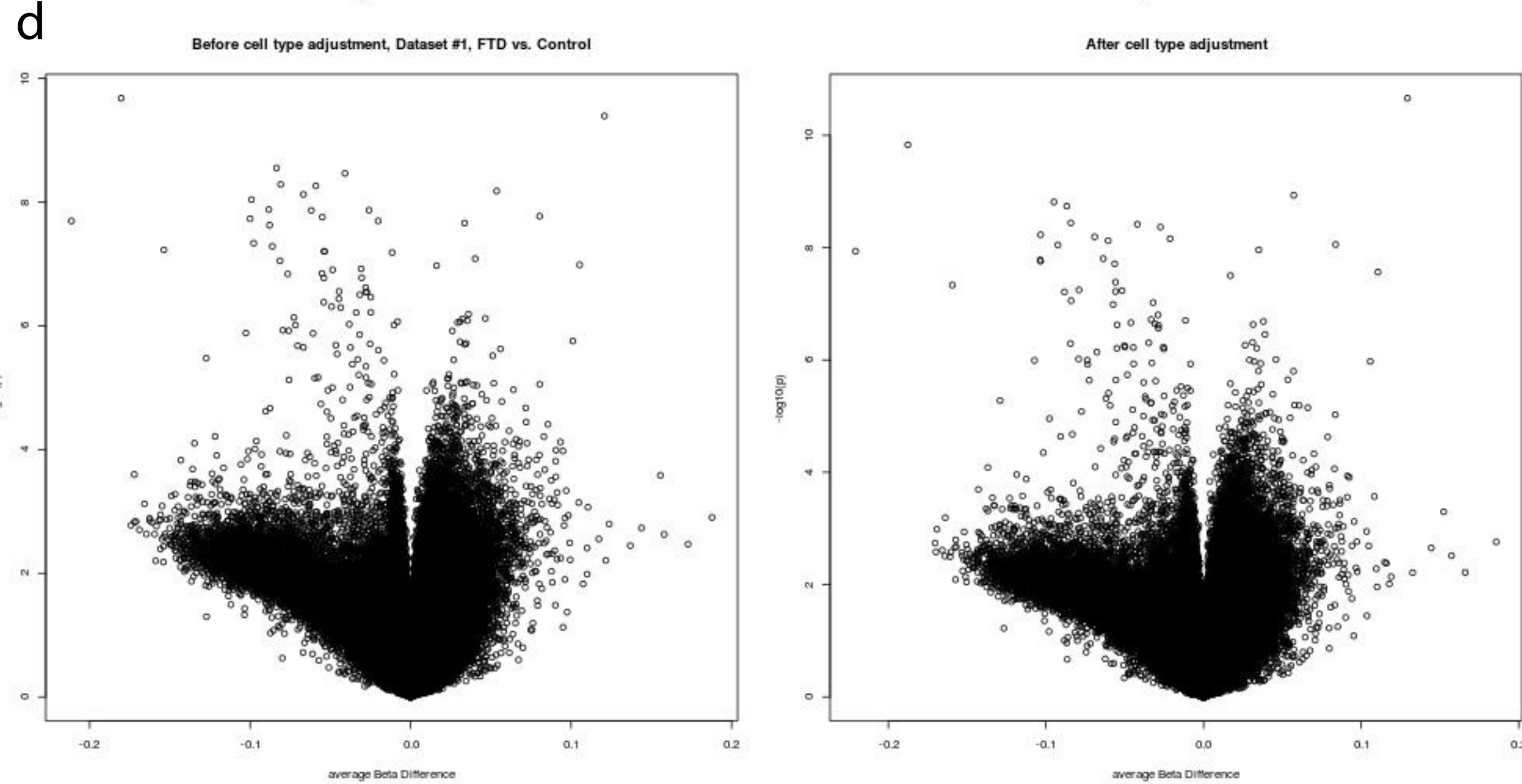

Supplement: Figure S11 — Volcano plots representing DMPs before and after cell type adjustment, in AD (a), PSP (b), FTD in dataset #1 (c), and FTD in dataset #2 (d). (PDF) [file pgen.1004211.s011.pdf]
