# Supplementary material for: An Epigenetic Signature in Peripheral Blood Associated with the Haplotype on 17q21.31, a Risk Factor for Neurodegenerative Tauopathy
Source: PLoS Genet. 2014 Mar 6;10(3):e1004211. doi: 10.1371/journal.pgen.1004211 (PMC3945475; doi:10.1371/journal.pgen.1004211)
Supplement: Table S1 — Demographic characteristics of the subjects enrolled in the study. (DOCX) [file pgen.1004211.s012.docx]

**Table S1**. Demographic characteristics of the subjects enrolled in the study.

| Dataset #1 | Control | FTD | PSP |
| --- | --- | --- | --- |
| Number of subjects | 93 | 55 | 40 |
| Caucasian (%) | 76.09 (n = 92) | 79.63 (n = 54) | 91.18 (n = 34) |
| Female (%) | 57.78 (n=90) | 45.83 (n=48) | 29.17 (n=24)* |
| Age at onset ± SD | - | 57.11±9.83 (n=46) | 63.52 ± 7.00 (n=29) |
| Age at study ± SD | 69.09±10.22 (n=93) | 65.60±10.16 (n=55)* | 70.36±7.44 (n=39) |
| APOE4 carriers (%) | 20.65 (n=92) | 25.45 (n=55) | 11.76 (n=17) |
| APOE4 frequency (%) | 11.96 (n=92) | 13.64 (n=55) | 5.88 (n=17) |
| 17q21.31 H1 carriers (%) | 95.65 (n=92) | 94.44 (n=54) | 100.00 (n=33) |
| 17q21.31 H1 frequency (%) | 79.89 (n=92) | 77.78 (n=54) | 96.97 (n=33)** |
| Dataset #2 | Control | FTD | PSP |
| Number of subjects | 92 | 73 | 3 |
| Caucasian (%) | 73.03 (n = 89) | 80.82 (n = 73) | 100 (n = 2) |
| Female (%) | 68.66 (n=67) | 50.00 (n=28) | 0.00 (n=1) |
| Age at onset ± SD | - | 60.49 ± 7.51 (n=41) | 62.00 ± 1.41 (n=2) |
| Age at study ± SD | 70.13±9.20 (n=92) | 65.23±7.89 (n=73)*** | 65.33±5.03 (n=3) |
| APOE4 carriers (%) | 15.38 (n=91) | 24.66 (n=73) |  |
| APOE4 frequency (%) | 9.34 (n=91) | 13.70 (n=73) |  |
| 17q21.31 H1 carriers (%) | 91.30 (n=92) | 97.18 (n=71) | 100.00 (n=2) |
| 17q21.31 H1 frequency (%) | 80.98 (n=92) | 86.62 (n=71) | 100 (n=2) |
| **Total** | **Control** | **FTD** | **PSP** |
| Number of subjects | 185 | 128 | 43 |
| Caucasian (%) | 74.59 (n = 181) | 80.31 (n = 127) | 91.67 (n = 36)* |
| Female (%) | 62.42 (n=157) | 47.37 (n=76)* | 28.00 (n=25)** |
| Age at onset ± SD | - | 58.70 ± 8.92 (n=87) | 63.42±6.78 (n=31) |
| Age at study ± SD | 69.61 ± 9.72(n=185) | 65.39 ± 8.90 (n=128)*** | 70.00 ± 7.37 (n=42) |
| APOE4 carriers (%) | 18.03 (n=183) | 25.00 (n=128) | 11.76 (n=17) |
| APOE4 frequency (%) | 10.66 (n=183) | 13.67 (n=128) | 5.88 (n=17) |
| 17q21.31 H1 carriers (%) | 93.48 (n=184) | 96.00 (n=125) | 100.00 (n=35) |
| 17q21.31 H1 frequency (%) | 80.43 (n=184) | 82.80 (n=125) | 97.14 (n=35)** |

FTD: Frontotemporal Dementia, PSP: Progressive Supranuclear Palsy. In parentheses the numbers of subjects for whom data were available. APOE and 17q21.31 haplotypes, ethnicity, and gender frequencies were compared using the chi-square test, age at study by Student's *t*-test. ***: p<0.001; **: p<0.01; *: p< 0.05

Ethnicity was determined based on SNP data and comparison with Hapmap data (1301 samples from Phase III) in 271 subjects, and was self-reported in 88 subjects.
