# Supplementary material for: An Epigenetic Signature in Peripheral Blood Associated with the Haplotype on 17q21.31, a Risk Factor for Neurodegenerative Tauopathy
Source: PLoS Genet. 2014 Mar 6;10(3):e1004211. doi: 10.1371/journal.pgen.1004211 (PMC3945475; doi:10.1371/journal.pgen.1004211)
Supplement: Table S3 — Breakdown of subjects by disease, and by H1/H2 genotype at the 17q21.31 locus. (DOCX) [file pgen.1004211.s014.docx]

Table S3. Breakdown of subjects by disease, and by H1/H2 genotype at the 17q21.31 locus

| **Dataset #1** | **AD** | **Control** | **FTD** | **PSP** |
| --- | --- | --- | --- | --- |
| H1H1 | - | 59 | 33 | 31 |
| H1H2 | - | 29 | 18 | 2 |
| H2H2 | - | 4 | 3 | 0 |
| **Dataset #2** | **AD** | **Control** | **FTD** | **PSP** |
| H1H1 | 9 | 65 | 54 | 2 |
| H1H2 | 5 | 19 | 15 | 0 |
| H2H2 | 1 | 8 | 2 | 0 |
| **Total** | **AD** | **Control** | **FTD** | **PSP** |
| H1H1 | 9 | 124 | 87 | 33 |
| H1H2 | 5 | 48 | 33 | 2 |
| H2H2 | 1 | 12 | 5 | 0 |
