# Supplementary material for: An Epigenetic Signature in Peripheral Blood Associated with the Haplotype on 17q21.31, a Risk Factor for Neurodegenerative Tauopathy
Source: PLoS Genet. 2014 Mar 6;10(3):e1004211. doi: 10.1371/journal.pgen.1004211 (PMC3945475; doi:10.1371/journal.pgen.1004211)
Supplement: Table S4 — R-squared coefficients from multivariate linear regression model for 3 the top PSP-related DMPs. (DOCX) [file pgen.1004211.s015.docx]

Table S4. R-squared coefficients from multivariate linear regression model for 3 top PSP-related DMPs.

|  | **cg23758822** | **cg22968622** | **cg12609785** |
| --- | --- | --- | --- |
| **Dx_Status** | 0.080** | 0.009 | 0.012 |
| **H1_freq** | 0.081** | 0.844*** | 0.602*** |
| **Ethnicity** | 0.003 | 0.001 | 0.009* |
| **AGE** | 0.002 | 0.001 | 0.000 |

* p< 0.05, ** p < 0.01, *** p < 0.001
