# Supplementary material for: An Epigenetic Signature in Peripheral Blood Associated with the Haplotype on 17q21.31, a Risk Factor for Neurodegenerative Tauopathy
Source: PLoS Genet. 2014 Mar 6;10(3):e1004211. doi: 10.1371/journal.pgen.1004211 (PMC3945475; doi:10.1371/journal.pgen.1004211)
Supplement: Table S5 — Significant p-value computed using double-bootstrap standard error. 1000 bootstrap iterations were used for each of the two bootstrap methods of standard error estimation. (DOCX) [file pgen.1004211.s016.docx]

**Table S5.** Significant p-values computed using double-bootstrap standard error. 1000 bootstrap iterations were used for each of the two bootstrap methods of standard error estimation.

|  | Dataset #1 | | | | Dataset #2 | | | |
| --- | --- | --- | --- | --- | --- | --- | --- | --- |
| Cell type | H1 dominant | H1 recessive | FTD | PSP | H1 dominant | H1 recessive | FTD | AD |
| CD8T | 0.295 | 0.462 | 0.043 | 0.097 | 0.047 | 0.001 | 0.071 | 0.014 |
| CD4T | 0.504 | 0.031 | 0.108 | 0.811 | 0.813 | 0.114 | 0.001 | 0.002 |
| NK | 0.824 | 0.096 | 0.014 | 0.065 | 0.019 | 0.002 | 0.004 | 0.537 |
| B-cell | 0.433 | 0.030 | 0.004 | 0.234 | 0.194 | 0.567 | 0.101 | 0.526 |
| Mono | 0.010 | 0.637 | 0.740 | 0.847 | 0.685 | 0.520 | 0.669 | 0.577 |
| Gran | 0.148 | 0.657 | 0.048 | 0.462 | 0.763 | 0.889 | 0.006 | 0.421 |

NK: natural killer, Gran: granulocytes, Mono: monocytes
