# Supplementary material for: An Epigenetic Signature in Peripheral Blood Associated with the Haplotype on 17q21.31, a Risk Factor for Neurodegenerative Tauopathy
Source: PLoS Genet. 2014 Mar 6;10(3):e1004211. doi: 10.1371/journal.pgen.1004211 (PMC3945475; doi:10.1371/journal.pgen.1004211)
Supplement: Table S6 — Breakdown of the 273 samples for which SNP array data and methylation data are available. (DOCX) [file pgen.1004211.s017.docx]

Table S6. Breakdown of the 273 samples for which SNP array data and methylation data are available.

| **Dataset #1** | **AD** | **Control** | **FTD** | **PSP** |
| --- | --- | --- | --- | --- |
| H1H1 | 0 | 50 (39) | 32 (27) | 8+ 6 rs1052335 inferred (7) |
| H1H2 | 0 | 24 (21) | 17 (16) | 1 (1) |
| H2H2 | 0 | 3 (3) | 3 (3) | 0 |
| **Dataset #2** | **AD** | **Control** | **FTD** | **PSP** |
| H1H1 | 9 (8) | 38 (29) | 41+ 2 rs1052553 inferred (34) | 0 |
| H1H2 | 4 (4) | 12 (12) | 13 (13) | 0 |
| H2H2 | 1 (1) | 7 (6) | 2 (2) | 0 |
| **Total** | **AD** | **Control** | **FTD** | **PSP** |
| H1H1 | 9 (8) | 88 (68) | 73 + 2 rs1052553 inferred (61) | 8 + 6 rs1052335 inferred (7) |
| H1H2 | 4 (4) | 36 (33) | 30 (29) | 1 (1) |
| H2H2 | 1 (1) | 10 (9) | 5 (5) | 0 |

In parentheses is the number of individuals of European descent (total n=226). Haplotype at 17q21.31 was called using rs1560310 in all samples except 8 (6 PSP, 2 FTD), for which rs1052335 was used.
