# Supplementary material for: An Epigenetic Signature in Peripheral Blood Associated with the Haplotype on 17q21.31, a Risk Factor for Neurodegenerative Tauopathy
Source: PLoS Genet. 2014 Mar 6;10(3):e1004211. doi: 10.1371/journal.pgen.1004211 (PMC3945475; doi:10.1371/journal.pgen.1004211)
Supplement: Table S8 — Methylation QTL analysis for 3 DMPs within 17q21.31 in in 226 individuals of European descent. (DOCX) [file pgen.1004211.s019.docx]

**Table S8.** Methylation QTL analysis for 3 DMPs within 17q21.31, in 226 individuals of European descent

| Probe | Number of associated SNPs | R-squared  (mean ± SD, range) | % within 17q21.31 |
| --- | --- | --- | --- |
| cg22968622 | 113 | 0.773 ± 0.159, 0.255~0.982 | 99.12 |
| cg17117718 | 113 | 0.727± 0.156, 0.264 ~ 0.8882 | 99.12 |
| cg19832721 | 106 | 0.605± 0.087, 0.258 ~ 0.719 | 99.06 |
