# Supplementary material for: An Epigenetic Signature in Peripheral Blood Associated with the Haplotype on 17q21.31, a Risk Factor for Neurodegenerative Tauopathy
Source: PLoS Genet. 2014 Mar 6;10(3):e1004211. doi: 10.1371/journal.pgen.1004211 (PMC3945475; doi:10.1371/journal.pgen.1004211)
Supplement: Table S10 — Methylation QTL analysis for 3 DMPs within 17q21.31 in 273 individuals. (DOCX) [file pgen.1004211.s021.docx]

**Table S10.** Methylation QTL analysis for 3 DMPs within 17q21.31

| Probe | Number of associated SNPs | R-squared (mean ± SD, range) | % within 17q21.31 |
| --- | --- | --- | --- |
| cg22968622 | 117 | 0.745 ± 0.22, 0.106~0.983 | 99.15 |
| cg17117718 | 115 | 0.717± 0.198, 0.2 ~ 0.893 | 99.13 |
| cg19832721 | 111 | 0.572± 0.134, 0.18 ~ 0.698 | 99.1 |
