# Supplementary material for: An Epigenetic Signature in Peripheral Blood Associated with the Haplotype on 17q21.31, a Risk Factor for Neurodegenerative Tauopathy
Source: PLoS Genet. 2014 Mar 6;10(3):e1004211. doi: 10.1371/journal.pgen.1004211 (PMC3945475; doi:10.1371/journal.pgen.1004211)
Supplement: Table S11 — Differentially methylated CpGs by genotype, found by reduced representation bisulfite sequencing. (DOCX) [file pgen.1004211.s022.docx]

**Table S11.** Differentially methylated CpGs by genotype, found by reduced representation bisulfite sequencing

| **Chromosome** | **Base** | **Position (hg18)** | **Context** | **p-value (uncorrected)** | **nearest gene** |
| --- | --- | --- | --- | --- | --- |
| chr10 | G | 74087808 | CG | 5.15E-12 | *MICU1* (intergenic) |
| chr17 | C | 41082844 | CG | 2.25E-12 | *CRHR1* (intronic) |
| chr17 | C | 41182407 | CG | 4.56E-12 | *CRHR1* (intronic) |
| chr17 | C | 41363555 | CG | 1.91E-12 | *MAPT* (intronic) |
| chr17 | G | 41641866 | CG | 1.77E-13 | *KIAA1267* (intronic) |
| chr17 | C | 52232615 | CG | 3.39E-09 | *C17orf67* (intronic) |
| chr17 | C | 52232671 | CG | 7.71E-09 | *C17orf67* (intronic) |
| chrX | C | 107632332 | CG | 1.35E-11 | *COL4A5* (intronic) |
| chrX | C | 107632359 | CG | 6.72E-11 | *COL4A5* (intronic) |
