# Supplementary material for: An Epigenetic Signature in Peripheral Blood Associated with the Haplotype on 17q21.31, a Risk Factor for Neurodegenerative Tauopathy
Source: PLoS Genet. 2014 Mar 6;10(3):e1004211. doi: 10.1371/journal.pgen.1004211 (PMC3945475; doi:10.1371/journal.pgen.1004211)
Supplement: Table S12 — NEO predictions for each identified haplotype-dependent DMP. (DOCX) [file pgen.1004211.s023.docx]

**Table S12:** NEO predictions for each identified haplotype-dependent DMP.

| **Methylated Site** | **Within Gene** | **LEO.NB score (mediation model)†** | **Predicted Causal Model‡** | **Predicted Model P value** | **Model RMSEA** |
| --- | --- | --- | --- | --- | --- |
| cg00846647 | MAPT | -1.68 | Meth<Hapl>PSP | 0.019 | 0.143 |
| cg04703951 | - | -0.104 | Meth<Hapl>PSP | 0.089 | 0.0932 |
| cg07870213 | DND1 | 0.378 | Hapl>Meth>PSP | 0.578 | 0 |
| cg16228356 | - | -2.44 | Meth<Hapl>PSP | 0.000 | 0.234 |
| cg17117718 | - | 0.85 | Hapl>Meth>PSP | 0.783 | 0 |
| cg18878992 | MAPT | -2.22 | Meth<Hapl>PSP | 0.001 | 0.211 |
| cg19832721 | KIAA1267 | -0.874 | Meth<Hapl>PSP | 0.089 | 0.0931 |
| cg22968622 | - | 1.51 | Hapl>Meth>PSP | 0.202 | 0.0537 |
| cg23955979 | - | -0.326 | Meth<Hapl>PSP | 0.081 | 0.0965 |

† LEO.NB score is defined as the base 10 logarithm of the ratio of p values for the mediation model and the next most likely causal model

**‡** NEO chooses between five causal models; here, the arrows indicate the direction of causality.
