# Supplementary material for: An Epigenetic Signature in Peripheral Blood Associated with the Haplotype on 17q21.31, a Risk Factor for Neurodegenerative Tauopathy
Source: PLoS Genet. 2014 Mar 6;10(3):e1004211. doi: 10.1371/journal.pgen.1004211 (PMC3945475; doi:10.1371/journal.pgen.1004211)
Supplement: Text S1 — Additional analyses including: 1) Testing the 17q21.31 haplotype effect in patients and controls separately; 2) Impact of estimated relative cell counts in peripheral blood; 3) Genome-wide methylation QTL analysis in the entire dataset (n = 273); 4) Differential methylation analysis using the combined dataset (n = 371 samples). (DOCX) [file pgen.1004211.s024.docx]

SUPPLEMENTARY MATERIAL

*Testing the 17q21.31 haplotype effect in patients and controls separately.* We tested the dominant model (i.e. H1 carriers vs. H2/H2) in (1) controls and (2) FTD patients separately, and the recessive model (i.e. H1/H1 carriers vs. the remaining samples) in (3) AD patients, due to the small number of AD patients who were H2/H2 carriers. We were not able to perform a separate analysis for PSP subjects, as they were almost exclusively H1/H1 carriers.

H1 carriers were compared vs. non-carriers in control samples only (92 samples in each dataset, table S3). 110 DMPs were identified in dataset #1, 9 of which were located in the 17q21.31 region (*p* = 2.605 × 10^-11^, hypergeometric test). In dataset #2, 26 DMPs were identified, 10 of which were located within the 17q21.31 region (p = 2.243 × 10^-20^, hypergeometric test).

We then compared H1 carriers vs. non-carriers in FTD samples only. Sixteen DMPs were identified in dataset #1 (n=55), 5 of which were within the 17q21.31 region (p = 2.697 × 10^-11^, hypergeometric test). Five were identified in dataset #2 (n=73), 2 of which were in 17q21.31 region (p = 5.883 × 10^-07^, hypergeometric test).

Finally, due to the small number of AD patients who were H2H2 carriers, we tested the recessive model on AD samples in dataset #2 (n=15). The only DMP (cg22968622) was located within the 17q21.31 region.

In conclusion, in all cases we observed an overrepresentation of DMPs located within the 17q21.31 region, further supporting the notion that the observed changes in methylation are mostly due to genotype differences rather than disease status.

*Impact of estimated relative cell counts in peripheral blood.* The difference of cell type distribution between case and control were evaluated using the method developed by Houseman et al. [1,2] and based on 500 loci whose methylation levels reflect the relative proportions of immune cells in unfractionated whole blood.

Briefly, we used methylation data available for 385 of the 500 loci (after quality control and probe removing). First, we estimated the blood cell type distribution for each sample using the methylation level of the 385 loci. Second, we applied a linear mixed-effect model considering (1) main blood cell types distribution as dependent variables; (2) disease status (or 17q21.31 haplotype), age, ethnicity, and gender as fixed effects; and (3) chip number as a random effect. We did not observe association of disease status or 17q21.31 haplotype with specific blood cell types (Table S5). Volcano plots (Figure S11) also showed no major differences in the number of DMPs when comparing before and after cell type adjustment.

*Genome-wide methylation QTL analysis in the entire dataset (n=273)*. We performed a methylation QTL (methQTL) analysis in a subset of 273 individuals for whom whole-genome SNP data was available (Table S6). We assessed association of genetic variants with methylation levels at 3 CpGs within 17q21.31 (cg22968622, cg17117718, cg19832721) in each dataset. We identified on average 113 genome-wide significant signals (Bonferroni-adjusted *p* ≤ 0.05), all located within the 17q21.31 region (Figure S4, Table S9). These variants accounted for a proportion of variability ranging between 10.6% and 98.3% (mean R-squared = 0.678, Figure S4, Table S10) further confirming that genetic variants at 17q21.31 are controlling methylation levels in *cis* in the same region.

*Differential methylation analysis using the combined dataset (n=371 samples).* After combining datasets #1 and #2, probes filtered in at least one dataset were removed, and the ComBat algorithm [3] was applied to remove batch effects. 66,877 SNP-containing probes were excluded from further analysis, resulting in 397,528 probes analyzed in 371 samples.

We first compared H1 carriers (genotypes H1/H1 and H1/H2) vs. H2/H2 (dominant model) and identified 34 differentially methylated probes (BH-adjusted *p* ≤ 0.05), 24 of which were located within the 17q21.31 region (p = 4.22 × 10^-53^, hypergeometric test). Sixteen of these probes were also identified in both datasets when they were analyzed separately.

Second, we compared H1/H1 subjects vs. H2 carriers (genotypes H1/H2 and H2/H2, recessive model) and identified 45 differentially methylated probes (BH-adjusted *p* ≤ 0.05), 36 of which were located within the 17q21.31 region (p = 2.19 × 10^-81^, hypergeometric test). Twenty-three of these probes were also identified in both datasets when they were analyzed separately. The 3 top DMPs (with an absolute aβD > = 0.1) identified in the two datasets when analyzed separately were also significantly differentially methylated in the combined analysis.

REFERENCES

1. Houseman EA, Accomando WP, Koestler DC, Christensen BC, Marsit CJ, et al. (2012) DNA methylation arrays as surrogate measures of cell mixture distribution. BMC Bioinformatics 13: 86. doi:10.1186/1471-2105-13-86.

2. Koestler DC, Christensen B, Karagas MR, Marsit CJ, Langevin SM, et al. (2013) Blood-based profiles of DNA methylation predict the underlying distribution of cell types: a validation analysis. Epigenetics 8: 816–826. doi:10.4161/epi.25430.

3. Johnson WE, Li C, Rabinovic A (2007) Adjusting batch effects in microarray expression data using empirical Bayes methods. Biostatistics 8: 118–127. doi:10.1093/biostatistics/kxj037.
