# Supplementary material for: An Epigenetic Signature in Peripheral Blood Associated with the Haplotype on 17q21.31, a Risk Factor for Neurodegenerative Tauopathy
Source: PLoS Genet. 2014 Mar 6;10(3):e1004211. doi: 10.1371/journal.pgen.1004211 (PMC3945475; doi:10.1371/journal.pgen.1004211)
Supplement: Figure S3 — Methylation QTL analysis in 226 individuals of European descent. (a) scatterplot representing the R-squared for each of the SNPs in the 17q21.31 region associated with at least one of the three DMPs. Gray: not significant SNPs. (b) corresponding genomic region at 17q21.31 (UCSC Genome Browser, hg19). Top significant SNPs controlling the three DMPs are highlighted in red. Age and the first two principal components generated from MDS analysis were added as covariates. (PDF) [file pgen.1004211.s003.pdf]

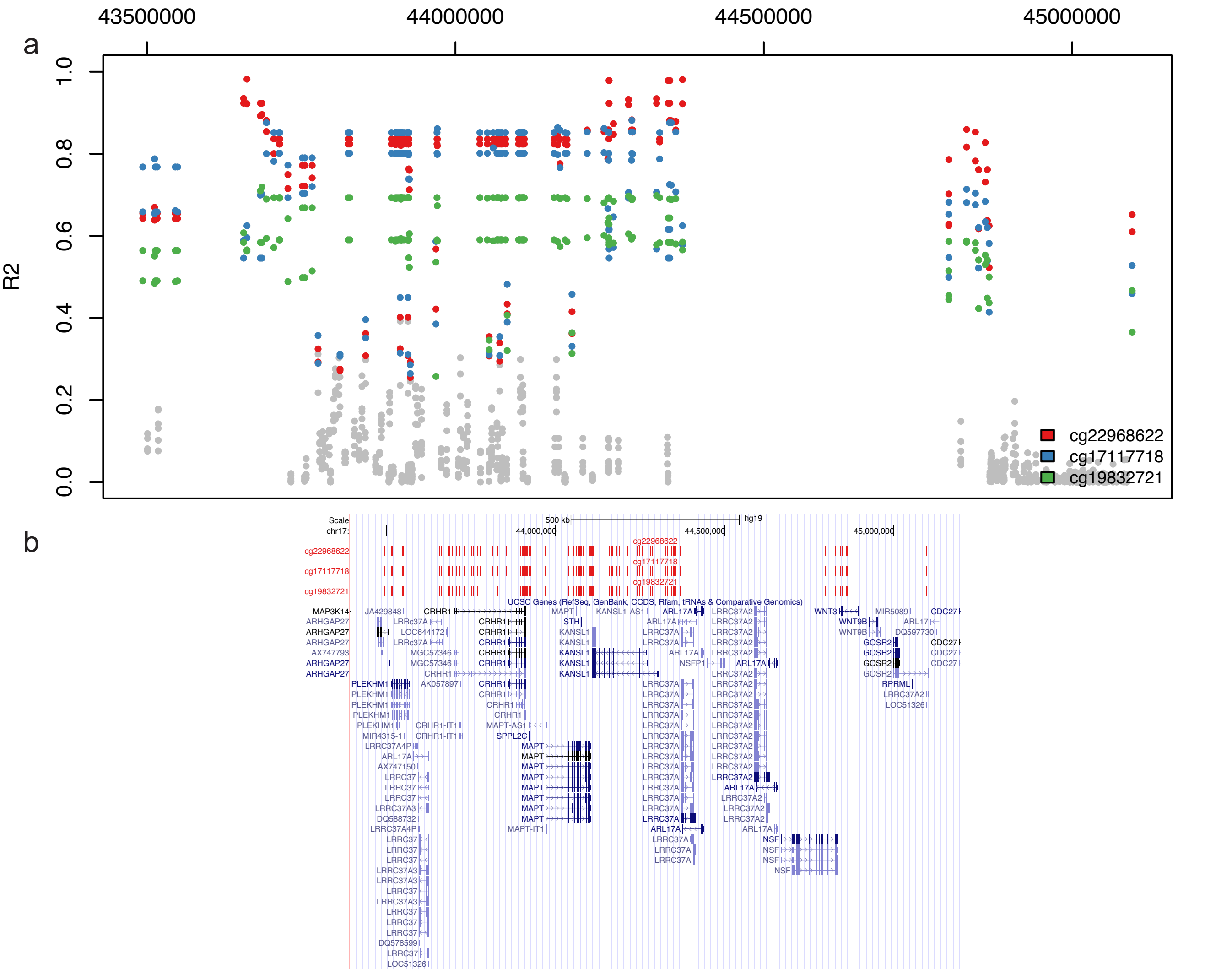
a
b
43500000
44000000
44500000
45000000
R2
0.0
0.2
0.4
0.6
0.8
1.0
cg22968622
cg17117718
cg19832721
Scale
500 kb
hg19
chr17:
44,000,000
44,500,000
45,000,000
UCSC Genes (RefSeq, GenBank, CCDS, Rfam, tRNAs & Comparative Genomics)
MAP3K14
ARHGAP27
AX747793
PLEKHM1
MIR4315-1
LRRC37A4P
ARL17A
AX747150
LRRC37
LRRC37A3
DQ588732
DQ578599
LOC51326
JA429848
LRRC37A
LOC644172
MGC57346
CRHR1
AK057897
CRHR1-IT1
MAPT-AS1
SPPL2C
MAPT
MAPT-IT1
STH
KANSL1
KANSL1-AS1
NSFP1
LRRC37A2
NSF
WNT3
WNT9B
MIR5089
ARL17
DQ597730
GOSR2
CDC27
RPRML
